# Improving State Government's Responsiveness to Family Planning Interventions in Nigeria Using an Innovative Reflection and Action Tool

**DOI:** 10.9745/GHSP-D-22-00189

**Published:** 2023-12-18

**Authors:** Lekan Ajijola, Victor Igharo, Nneoma Anieto, Lisa Mwaikambo

**Affiliations:** a The Challenge Initiative, Nigeria Hub, Johns Hopkins Center for Communication Programs, Abuja, Nigeria.; b The Challenge Initiative, Johns Hopkins Center for Communication Programs, Baltimore, MD, USA.

## Abstract

**Background:**

The Challenge Initiative (TCI) works with state governments in Nigeria to scale high-impact family planning and reproductive health (FP/RH) interventions rapidly and sustainably. The Reflection and Action to Improve Self-reliance and Effectiveness (RAISE) tool is an innovative responsive feedback (RF) mechanism developed by TCI to periodically monitor governments' readiness to sustain implementation of their FP/RH interventions.

**RAISE Description:**

The RAISE tool—a facilitated, self-administered tool used quarterly to track program improvements, identify gaps, and provide feedback—contains FP/RH progress indicators across 4 pillars: political and financial commitment, government capacity, institutionalization, and sustained demand. As a maturity matrix that helps state governments to understand the extent of their self-reliance in leadership and program management, the tool measures the stages of capacity: “beginning” (54% and below), “developing” (55–69%), “expanding” (70–84%), and “mature” (85% and above). Participants self-administer the tool with a trained government facilitator, develop a remediation plan with timelines and responsible persons assigned to address identified gaps, and communicate the plan to the broader FP/RH program team.

**Assessment Results:**

Thirteen supported states have completed 5 rounds of assessments between June 2020 and September 2022. Baseline results revealed that 4 states were at the developing stage, 8 were at the expanding stage, and 1 had a mature program. The most recent assessment revealed mature capacity for 9 states while the 4 others are in the expanding stage. Consequently, all the states demonstrated improved government self-reliance over the course of the year.

**Conclusion:**

To ensure objectivity, RAISE participants, who include government policymakers, program managers, and implementers, use available government data sources to complement assessment findings. As an RF mechanism, the RAISE tool contributed to governments' enhanced leadership and management of their FP/RH programs, with current considerations for modifications to serve other primary health care programs.

## Abstract (precis)

Use of a responsive feedback tool improved government-led family planning programming by enabling reflection, monitoring of capacity-building, and action planning.

## Teaser

**Key Findings:**

- The Challenge Initiative (TCI) Nigeria applied the Reflection and Action to Improve Self-reliance and Effectiveness (RAISE) tool as a responsive feedback mechanism to enable all 13 state governments to assess their progress toward self-reliance in family planning/reproductive health programming; identify gaps; and incorporate learning and feedback to make improvements throughout the implementation life cycle.
- Adaptation of the RAISE tool to Nigerian and state contexts improved local and state government teams' understanding of adaptive program management, leading to positive changes.

**Key Implications:**

- Policymakers should adopt and institutionalize the RAISE tool for responsive feedback to make improvements in primary health care programs in real time.
- Government program managers and implementers should scale up use of the RAISE tool to regularly obtain and analyze feedback data for continuous improvement.
- Decision-makers should use the findings from the RAISE assessments to make evidence-based decisions for adaptive management of their health programs.

## BACKGROUND

Nigeria is undergoing rapid urbanization with a growing population that is especially concentrated in urban centers. At the current growth rate of about 2.8%–3.0% a year, Nigeria's urban population is expected to double over the next 2 decades.^1^ Health outcomes of urban women living in poverty are considerably worse than those of their wealthier urban counterparts and, in some cases, of rural women.^2^^–^^5^ Although women in urban areas have fewer children (4.5 children per woman) compared to those in rural areas (5.9 children per woman), the fertility rate is impacted by education and wealth quintile in Nigeria. For example, urban poor women in Nigeria often experience similar fertility rates as women in rural areas.^6^

As a result, it is essential to reach urban women and girls of reproductive age, particularly those living in poor urban settlements, with health and family planning (FP) services and to empower them to decide and act freely about whether and when to have children. Contraceptive use improves women's and children's health in many ways, including reducing maternal mortality risks and improving child survival.^7^ There is a solid evidence base for what works in FP programming.^8^^,^^9^ Despite this knowledge, evidence-based interventions have not been effectively scaled widely, including in Nigeria. The Federal Ministry of Health set a goal to improve the modern contraceptive prevalence rate (mCPR) to 27% by 2020; in 2018, the mCPR was 17% among currently married women aged 15–49 years, and unmet need for FP among currently married women was 19%.^6^ In addition, mCPR was lowest among poor women: 4% among those in the lowest wealth quintile compared to 22% among the highest quintile.^6^ Considering that Nigeria did not meet this set target by 2020, a review of progress and restructure of a timeline for targets was conducted as part of the Nigeria FP2030 commitment, with a renewed commitment to achieve 27% mCPR by 2030.^10^

Ownership and sustainability of health programs remain challenges in low- and middle-income countries, including Nigeria, with local governments depending heavily on international donors and partners for support. Challenges in Nigeria include inadequate domestic funding for FP programs, limited human resource and monitoring and evaluation capacity, and weak health systems.^11^^–^^15^ State governments in Nigeria have the autonomy to decide how to manage their health budget annually; prioritization of FP in those budgets varies between states depending on other competing priorities, government capacity, and program implementation. Suboptimal state FP program response is further compounded by weak government capacity to institutionalize and scale up proven FP interventions.

Ownership and sustainability of health programs remain challenges in Nigeria.

To address these challenges, it is imperative that Nigerian state governments and local government authorities actively lead and coordinate their family planning and reproductive health (FP/RH) programs. Traditionally, global health programming has been top-down, driven by major donors and governments of high-income countries.^16^ However, the 2005 Paris Declaration on Aid Effectiveness, followed by the Accra Agenda for Action (2008) and the Busan Agenda (2011), underscore the importance of country “ownership,” increased reliance on “country systems,” and “enhanced developing country capacities” tailored to country-specific situations and needs for development effectiveness.^17^ Both the Paris Declaration and the Accra Agenda included recommendations that technical assistance (TA) providers make capacity-building a fundamental feature of TA to enable countries to operate their health systems effectively and autonomously in the future,^18^ while the Busan Declaration placed explicit emphasis on learning as 1 of the core aspects of enhanced capacity.^17^

Local government ownership and capacity strengthening that is tailored to local needs is at the heart of The Challenge Initiative (TCI)'s “business unusual” approach, as outlined in Mwaikambo et al.^2^ TCI is an urban FP/RH program with a goal of achieving greater local government self-reliance to scale up evidence-based FP and adolescent and youth sexual and reproductive health (AYSRH) interventions, leading to sustained improvements in FP service delivery and increased use of modern contraception, especially among the urban poor. Funded by the Bill & Melinda Gates Foundation, Bayer AG, Comic Relief, and others, TCI provides coaching and technical and financial assistance to state governments to enable them to lead implementation of evidence-based FP and AYSRH interventions and ultimately sustain the positive outcomes of a government-driven FP/RH program.^19^

TCI puts state government leadership at the center of implementing, managing, and monitoring the proven FP and AYSRH interventions. States self-select to become a part of TCI, bringing their own financial and human resources to bear in scaling the proven interventions with the knowledge of their graduation from direct TCI support within 3–4 years. Led globally by the Bill & Melinda Gates Institute for Population and Reproductive Health (Gates Institute) and implemented by the Johns Hopkins Center for Communication Programs in Nigeria, TCI partnered with 13 states in its first phase, including Ogun, Delta, Kano, Niger, Bauchi, Anambra, Plateau, Taraba, Abia, Rivers, Nasarawa, Lagos, and Gombe states.

While the overarching purpose of TCI is to rapidly meet women's and couples' unmet need for modern contraception, it aims to effect changes in the health system along the way to ensure the results are sustainable, even after a state graduates from TCI. Therefore, in addition to measuring scale-up in the traditional sense of the number of states engaged and population reached, TCI developed the Reflection and Action to Improve Self-reliance and Effectiveness (RAISE) tool, which monitors a state's progress toward achieving sustainable scale-up of high-quality FP programming and outcomes in the following areas.

TCI's RAISE tool monitors a state's progress toward achieving sustainable scale-up of high-quality FP programming and outcomes.

- Increased political and financial commitment for FP among local government
- Capacity strengthening of local government stakeholders at the systems, organizational, and individual levels
- Institutionalization of TCI high-impact interventions and processes
- Sustained demand for FP services, including increased agency of women and supportive community norms

TCI designed RAISE as a responsive feedback (RF) tool based on empowerment evaluation theoretical principles and successful experiences in implementing organizational capacity assessment tools, with the intention to go beyond measuring and monitoring capacity development to institutionalizing a process for organizational reflection, learning, and problem-solving.^20^^–^^23^ Through quarterly assessments, state government teams reflect upon the progress and challenges encountered in financing, implementing, and managing their FP programs and ultimately arrive at actionable feedback and steps to ensure the program stays on track and achieves its intended outcomes. RAISE also fosters best practice sharing and enables state governments to lead and facilitate implementation of their improvement plans. The RAISE tool is used by state governments in partnership with TCI, therefore increasing the likelihood that it will be sustained beyond TCI's engagement period. It is currently being used by all 13 TCI-supported states in Nigeria as well as 97 TCI-supported cities across East Africa, Francophone West Africa, India, and the Philippines.^24^ The aim is that state and city governments will continue to use this RF tool and process themselves following graduation.

This article aims to investigate how RAISE has been used as an innovative RF mechanism to periodically evaluate a government's readiness to lead and implement evidence-based FP and AYSRH interventions. The tool was developed to help TCI explore the following questions: (1) How can TCI and government determine when a state is ready to graduate from TCI's direct support? (2) How can TCI effectively monitor a state's capacity to finance, implement, and monitor the outcomes from evidence-based FP and AYSRH interventions, with incorporation of feedback to adapt the program for improvement in real time? In this article, we review the RAISE assessment findings in Nigeria and explore trends in use of the RAISE tool for RF to improve FP/RH programs. We also reflect upon lessons learned from conducting 5 rounds of assessments with the tool and highlight adaptations in its use, as well as successes and ongoing challenges.

## RAISE TOOL

RAISE is a management and organization tool that uses an RF approach to assess and improve state government's self-reliance in FP/RH programming. RF is an integrated framework that incorporates elements of feedback, continuous learning, and engagement, premised on using foresight and learning to iterate programs for better results. The RAISE approach encompasses and incorporates the 5 key elements of RF in its implementation (Box 1).

BOX 1 Responsive Feedback Key Elements Incorporated in the Reflection and Action to Improve Self-reliance and Effectiveness (RAISE) Approach

1. **Requires diverse stakeholders to work together**. The RAISE tool's structured and participatory process allows state government teams across relevant departments to come together and self-assess their family planning/reproductive health program.
2. **Interrogates a program's theory of change (TOC) and makes assumptions explicit**. The Challenge Initiative's (TCI's) TOC aims to increase modern contraception through government-led and government-driven programming. The TOC enabled the TCI team to use responsive feedback to identify areas of testing, which formed the domains captured in the RAISE tool.
3. **Prioritizes information gaps where a program would benefit from data and course correction**. The RAISE tool enables government teams to track implementation progress, note gaps in service delivery, use data to corroborate findings collected, and make effective and evidence-informed decisions to improve their program.
4. **Seeks evidence to fill information gaps using learning questions**. The tool makes provision for documenting evidence for findings reported, which enables the assessment teams to continue to update learning questions to interrogate the gaps and identify corrective measures for program improvement.
5. **Advocates pause-and-reflect sessions to evolve a program based on evidence**. Following the RAISE assessment, the lead government facilitator convenes a feedback session in the form of a pause-and-reflect meeting, where relevant government staff and TCI reflect upon the findings and evidence and agree on action plans to improve programming.

**FIGURE 1:**
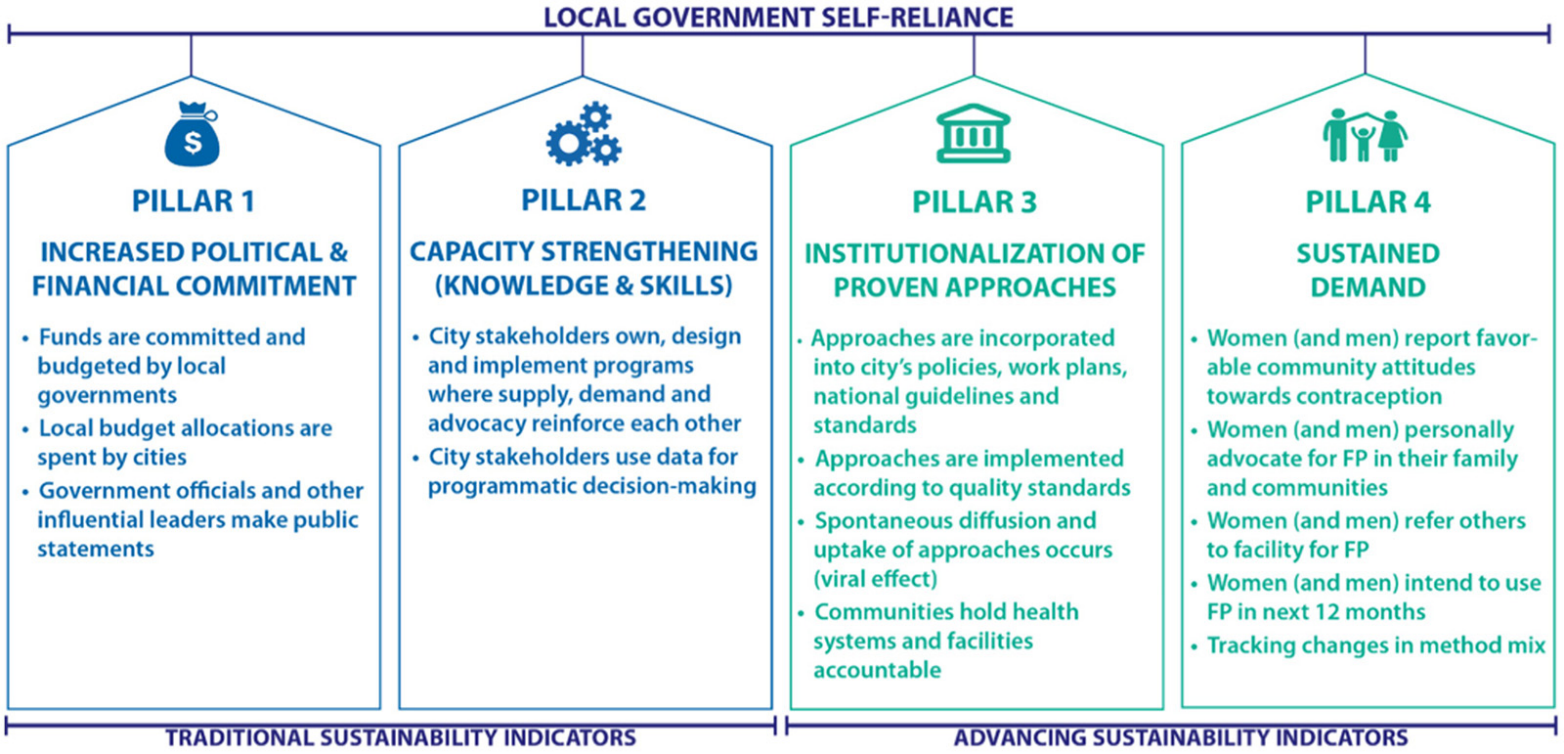
RAISE Sustainability Pillars Abbreviations: FP, family planning; RAISE, Reflection and Action to Improve Self-reliance and Effectiveness. Source: The Challenge Initiative.

The RAISE tool comprises 4 pillars of measurement: (1) political and financial commitment, (2) capacity strengthening, (3) institutionalization of proven approaches, and (4) sustained demand (Figure 1). Each domain has indicators that the assessors score from 1 (minimum) to 4 (maximum). The domains are weighted percentages to allow for easy aggregation of final scores.

In June 2020, TCI Nigeria adapted the global TCI RAISE tool to suit the country's context and respond to key issues limiting government self-reliance in FP/RH programming, specifically noting the challenge related to limited health expenditure and, more specifically, domestic financing of FP/RH programming. This adaptation process was done at the national level in Abuja in consultation with the TCI state and government teams to ensure that the state-specific context was considered while standardizing the indicators and assessment approach. This adaptation occurred at 2 levels: (1) an initial joint review of the tool by the state government and state TCI team members, and (2) a second review between the TCI teams at the state and national levels to aggregate feedback and considerations from the state teams. Consensus from the review of the tool led to weighting the 4 domains as follows, resulting in a total possible score of 100% for the Nigeria RAISE assessment.

TCI Nigeria adapted the global RAISE tool to suit its context and respond to key issues limiting government self-reliance in FP/RH programming and the challenge related to domestic financing of FP/RH programming.

- Domain 1, political and financial commitment: 30%, was deemed the most important by the government to achieve self-reliance in programming based on current realities.
- Domain 2, government capacity: 20%, was reported to be the least important considering the number of trainings and capacity-building exercises that have happened at the state level, with states having moderately acceptable numbers of trained FP providers.
- Domain 3, institutionalization of the proven interventions: 25%, was given equal importance as domain 4 and retained its original weight.
- Domain 4, sustained demand: 25%, was given equal importance as domain 3 and retained its original weight.

**FIGURE 2:**
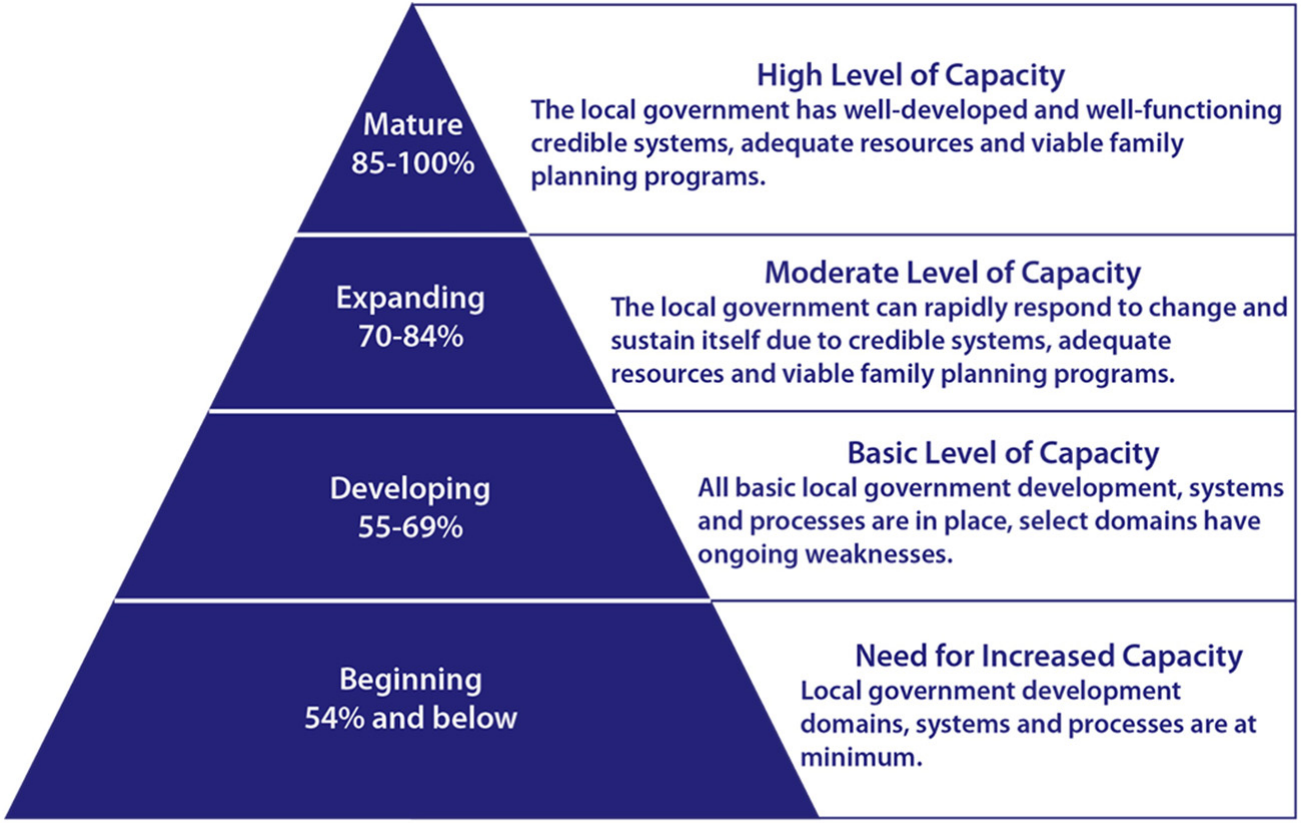
RAISE Ranking Levels Abbreviation: RAISE, Reflection and Action to Improve Self-reliance and Effectiveness. Source: The Challenge Initiative.

However, the Nigeria hub maintained the original overall categories of government maturity or capacity levels based on the aggregated final score (Figure 2). This categorization was originally developed in the global tool by a team of experts who initially piloted the tool, including members from the Gates Institute, TCI East Africa, and government teams from Kenya and Uganda. The categories of state government maturity stages toward self-reliance are as follows.

- “Beginning” (54% and below), showing increased capacity needed by government
- “Developing” (55%–69%), depicting basic government capacity
- “Expanding” (70%–84%), depicting moderate government capacity
- “Mature” (85% and above), showing a mature FP/RH program owned and led by government

TCI uses the RAISE tool to gauge state governments' self-reliance of their FP/RH programs and as 1 indicator among 2 others to measure state governments' progress toward graduation from the TCI program and its direct support. The 2 other graduation indicators, alongside a mature stage of RAISE (85% and above), include level of government funding for FP/RH programs and modern contraceptive uptake trends, as described by Finkle et al.^19^

Implementation of the RAISE assessment is coordinated and led by a designated government staff member in the FP/RH unit, usually the state FP coordinator, who ensures that relevant government staff is available to participate in the assessment. Critical to the assessment is the use of government data sources to complement and validate the scores given to the indicators measured in the RAISE tool. Some of the data sources used include the health management information system (HMIS) for review of the reproductive, maternal, newborn, and child health indicators; program data related to the proven interventions being implemented; and financial reports to assess the level of domestic funding. The HMIS data are reviewed for the quarter being assessed against the indicators in the RAISE tool to confirm if there was an increase or a dip in the number of new FP acceptors. Similarly, the state annual operational plans are reviewed for scoring domain 3—institutionalization of proven interventions—to assess the number of proven interventions adopted for the reporting quarter. The state TCI and government teams use a funding tracker to monitor the funding amount released by the state government and TCI to support program implementation on a quarterly basis. The government assessors develop an action or remediation plan to address suboptimal areas, with responsible persons identified to lead the corrective actions planned and set timelines for implementing the action plan. TCI typically participates in the first few assessments to provide coaching and guidance on the use of the tool before states fully take over the process. The government's leadership of this process is critical to its ownership of the actions associated with learnings that emerge from the assessment reflections and discussions.

Critical to the RAISE assessment is the use of government data sources to complement and validate the scores given to the indicators measured in the RAISE tool.

After adapting the tool, TCI Nigeria subsequently conducted an orientation for all TCI staff to ensure uniform understanding of the tool and seamless rollout across the supported states. TCI teams across the supported states, in turn, developed a RAISE implementation schedule that articulated quarterly assessment plans working with relevant government teams. Using the guidance provided in the tool, the TCI state teams worked with their state government counterparts to identify relevant participants for the RAISE assessment. The assessment teams, which include FP/RH policymakers, program managers, implementers, and implementing partners, differed slightly in composition based on the state context. These sets of participants are the target for adaptive management of health programs due to the nature of their work, which encompasses provision of TA and implementation of proven FP/RH interventions. The program managers and implementers work closely together to ensure that remediation plans from the assessments are implemented to cause the desired change and improvement.

### The RAISE Assessment Process

**FIGURE 3:**
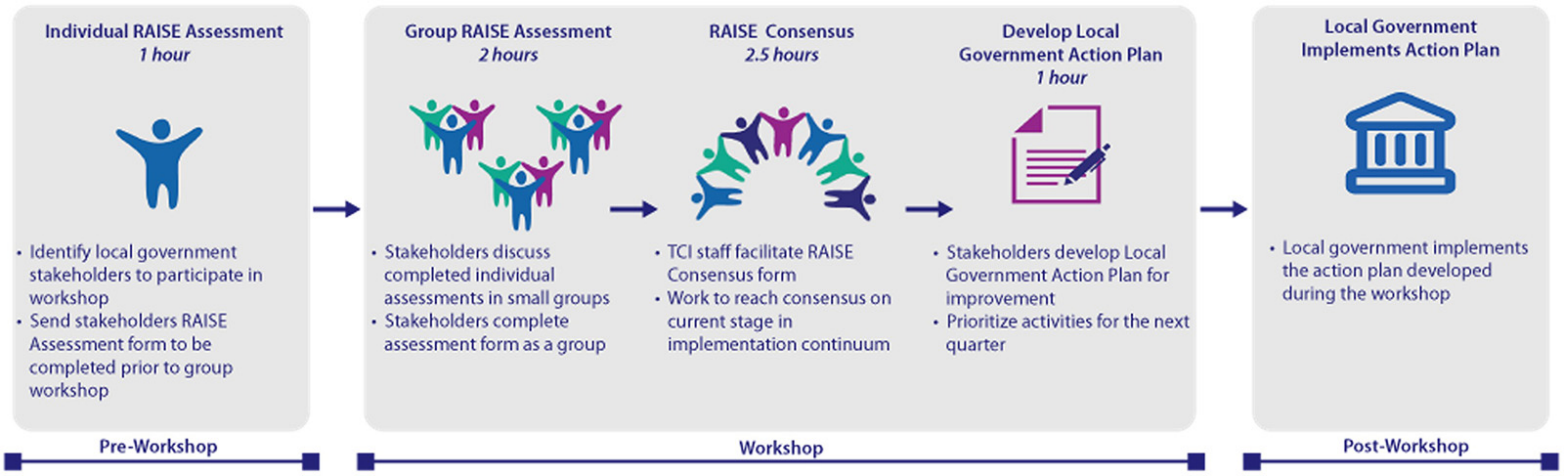
RAISE Assessment Process Abbreviation: RAISE, Reflection and Action to Improve Self-reliance and Effectiveness. Source: The Challenge Initiative.

The RAISE assessment is conducted on a quarterly basis using a multistep process (Figure 3).

**Pre-workshop review.** Individual participants listed in Box 2 are asked to review and familiarize themselves with the RAISE checklist (Supplement) ahead of the assessment workshop and come to the workshop with questions on areas or sections that are unclear. This step is skipped for future assessments except when there are new participants.

BOX 2 Participants in the Reflection and Action to Improve Self-reliance and Effectiveness (RAISE) Assessment Teams in Nigeria

**Policymakers**

- Honorable commissioner for health
- Executive secretary, State primary health care development agency
- Director, Public health
- Director, Planning, research, and statistics
- Director, Community services

**Program managers and implementers**

- State reproductive health coordinator
- State family planning coordinator
- State health educator
- State monitoring and evaluation officer
- State logistics officer
- Adolescent health desk officer
- State finance focal person

**Partners**

- The Challenge Initiative team
- Representatives from family planning/reproductive health implementing partners

**Small group assessment.** Participants are divided into small groups to build consensus on scoring the checklist indicators based on available evidence from the data sources mentioned earlier.

**Group workshop.** The small groups convene as 1 large group to review the small-group scores using available evidence and come to a consensus on the final scores per domain for the state.

**Local government action plan development.** The team jointly develops an action or remediation plan—including corrective actions, responsible persons, and timelines (usually within 2 months to allow for implementation before the next quarterly assessment)—to address gaps identified during the assessment.

The assessment team jointly develops an action or remediation plan to address gaps identified during the assessment.

The workshop is facilitated by a trained state government staff member, usually the state FP coordinator, with TA from the TCI state team. This facilitator works with the assessment team members to plan and conduct a dissemination and feedback meeting with the extended FP/RH team across relevant ministries, departments, and agencies while working together to implement the solutions proffered in the action plan. The RAISE assessment process is not complete until the assessment team has provided feedback on their findings and planned actions to relevant policymakers and other technocrats who will support the implementation of corrective actions to close identified gaps. The feedback summarizes components for which the state is performing optimally but focuses more on the challenges or issues leading to low scores that need to be addressed.

Policy and funding issues requiring attention are assigned to relevant directors who are part of the assessment team. The directors advocate for policy change or enforcement with the executive secretaries of the state primary health care development agencies and honorable commissioners for health of the state ministries of health and secure additional funding for the FP program when necessary. Once these changes are approved by the policymakers and translated into action, program managers and technocrats are duly notified, enabling them to implement the policies across implementing geographies in the state and request funds for relevant activities. Implementation of these interventions with attendant results empowers them to adjust the RAISE scores upward during the next assessment based on guidance.

Technical and quality-related gaps are assigned to program managers or technical leads on the assessment team to facilitate planned corrective actions. Examples of these gaps include the lack of FP supportive supervision systems, inadequate capacity of health providers to administer long-acting contraceptive methods, provider bias, poor documentation of services provided, and weak commodity logistics systems. To address the challenges and contribute to improved scores in subsequent assessments, the team organizes and includes quarterly FP supportive supervision in their workplans and plans coaching and mentoring sessions for providers in local government areas reporting gaps in service, commodities, and documentation, using available domestic funding and leveraging partner funding. Implementation of the action plan items on the remediation plan before the next assessment usually leads to improvement in the RAISE scores, thus moving the state toward program maturity. However, states are not always able to make equal progress on all items noted in the remediation plans before the next assessment. As a result, the items that are not completely resolved move to the following quarter's remediation plan.

The assessment team uses a tracker to measure progress and make further course corrections based on quarterly assessments implemented. TCI Nigeria also maintains a digital RAISE dashboard at the national level that tracks progress across all supported states. This allows TCI Nigeria to see at a glance how states are performing and follow up with the assessment teams to provide TA to any states with stagnant indicators. This is an important way in which the RAISE tool is used as an RF approach to elicit real-time improvement in FP/RH programming.

TCI Nigeria uses a digital RAISE dashboard as an RF mechanism to monitor performance across all states and follow up with teams to elicit real-time improvements in FP/RH programming.

## RESULTS

At the time of this publication, TCI Nigeria has supported implementation of the RAISE assessment across 13 states, with 5 rounds of assessment per state between June 2020 and September 2022, based on the start of their engagement with and graduation from TCI. The first group of states—Ogun, Delta, Kano, Niger, and Bauchi—began their engagement with TCI in 2018; these states graduated from direct support from TCI in June and September 2021. The second group of states—Anambra, Plateau, Rivers, Abia, and Taraba—began their engagement with TCI in 2019 and the last group—Nasarawa, Lagos, and Gombe—in 2021. The Supplement provides score details by state and time of TCI engagement.

Baseline RAISE assessments from the 13 states revealed that 4 states had a developing (stage 2) program, 8 states had an expanding (stage 3) program, and 1 state had a mature (stage 4) program. The baseline scores for the first 2 groups of states were likely influenced by the coaching and TA that the states received upon engaging TCI before the introduction of the RAISE tool in June 2020.

**FIGURE 4:**
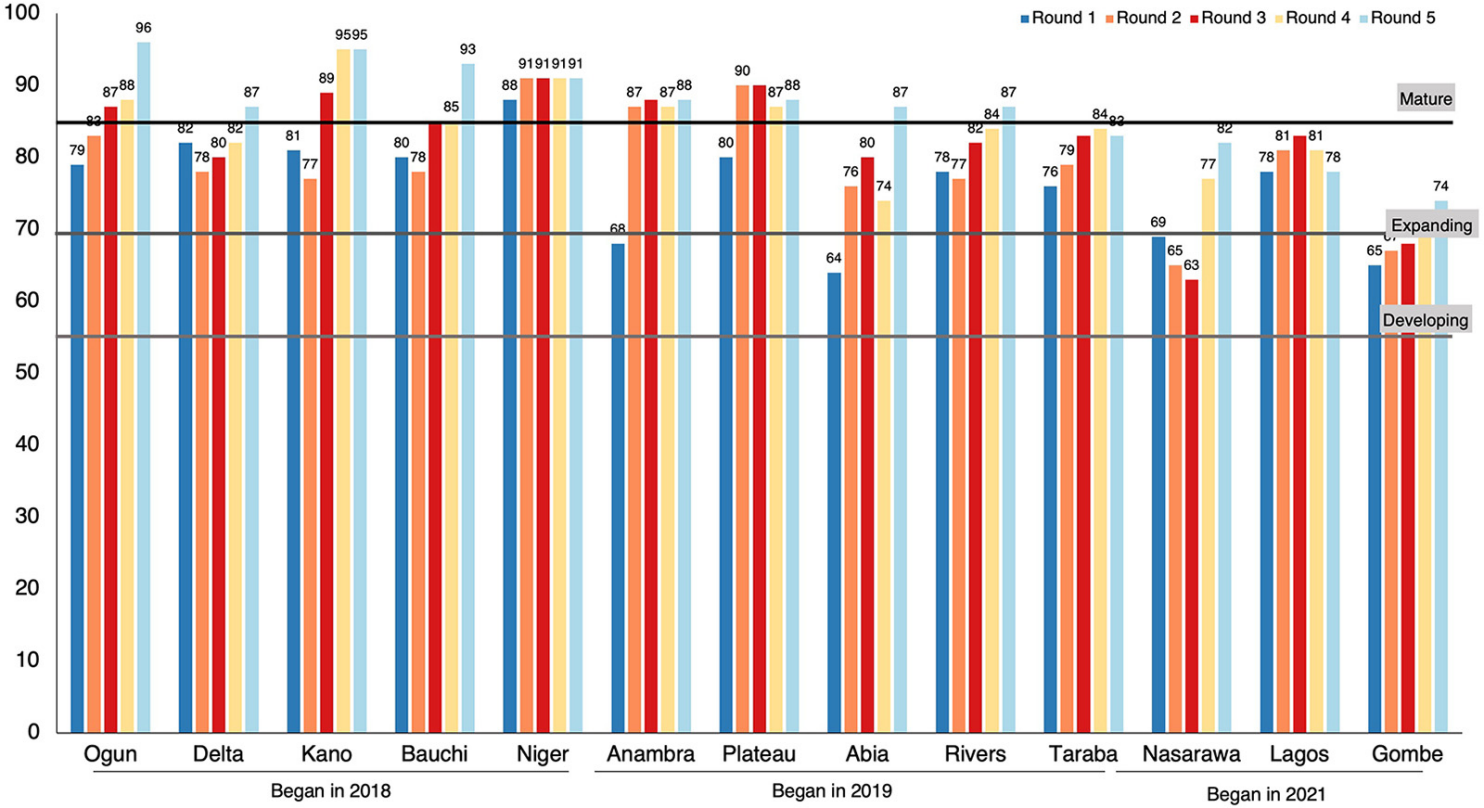
RAISE Assessment Implementation Scores Across 5 Rounds in 13 States in Nigeria, 2020–2022 Abbreviation: RAISE, Reflection and Action to Improve Self-reliance and Effectiveness.

Among the first group of 5 states that had the earliest engagement with TCI, all 5 states started at an acceptable stage of maturity, with 4 states in the expanding stage and 1 in the mature stage. This is consistent with TCI's expectations that these states would be ready to graduate from direct support within 3 to 4 years of engaging with TCI. By the third assessment round, 3 more states had moved into the mature stage, leaving only 1 state in the expanding stage of capacity that moved to mature stage by the fifth round (Figure 4).

Baseline assessments for the second group of 5 states that started engaging with TCI in 2019 revealed 2 at the developing stage and 3 at the expanding stage. Interestingly, 2 states moved into the mature stage by the second round of the assessment, and this increased to 4 states by the fifth round (Figure 4). These states graduated from TCI's direct support in April and May 2022.

The 3 states in the third group started their engagement with TCI in 2021. Among the 3 states, 2 states were at the developing stage at baseline, with the remaining 1 at the expanding stage. By the end of the fifth round, the 2 states with baseline developing stage had moved to the expanding stage of maturity. Figure 4 shows the composite scores for up to 5 rounds of assessments across all 13 states.

Application of the RAISE tool enabled state governments to self-assess their progress toward self-reliance in FP programming through regular analysis of findings and feedback data from the assessment, develop action plans to close identified gaps, and implement those plans to make improvements throughout the life cycle of the program. Assessment findings provided the basis for RF upon which evidence-based decisions were made by decision-makers and implementers to respond to emerging issues and challenges, leading to adaptive improvement.

Assessment findings provided the basis for RF upon which decision-makers and implementers made evidence-based decisions to respond to emerging challenges, leading to adaptive improvement.

## LESSONS LEARNED

The use of the RAISE tool as an RF mechanism has enabled each TCI-supported state to function as a learning organization, intentionally pausing quarterly to assess progress and identify challenges to program implementation. It has required a certain level of adaptation to the Nigerian context and nuance in interpreting the results. Buy-in of key government counterparts regarding the use of the RAISE tool and development of a RAISE implementation schedule enabled state teams to have relatively uninterrupted RAISE assessments quarterly. Assessment findings are triangulated with other sources of data, including data from the government's HMIS and program records data, to ensure objectivity of scores assigned.

Joint development of the action plan and the provision of feedback from assessment findings to key government policymakers are critical components of the RAISE process, enabling policymakers to make commitments and act. Policymakers have acted to enforce policies and secure funding because of these RAISE feedback sessions.

Supporting state and local government teams to own the RAISE tool by contributing to its adaptation to the Nigerian context and leading its implementation on a quarterly basis has improved their understanding of adaptive program management and led to numerous positive changes. This is reflected in the following comments from various government team members.

*I appreciate The Challenge Initiative for this assessment process, and I have come to realize that the assessment has kept us on track with regards to FP and the development of the remediation/action plan has assisted us in also tracking the level of implementation of our AOP [annual operational plan] with particular emphasis to the FP component. I want to recommend that similar assessment checklist should be adopted for use in other areas of health interventions like malaria, nutrition, etc.* —Permanent Secretary, Taraba State Ministry of Health

*The first assessment was an eye opener to the state in areas that require strong advocacy, such as budget for adolescent health and increase funding for FP.* —Head of Program Unit, Ogun State Primary Health Care Development Agency

*I initially thought of the RAISE process as cumbersome; however, as we progressed with the assessment seeing the final product, I was very impressed by the expository nature of the findings. They were succinct and empirical, and RAISE is indeed an eye opener. We will need TCI's support to adopt this tool for other [health] programs.* —Head of Planning, Research & Statistics, Anambra State Ministry of Health

*The things we knew are what we were working on before, but now, we know more on areas that really require our attention, and this has improved the way we work. RAISE has opened our eyes.* —State FP Coordinator, Ogun State Primary Health Care Development Agency

Supporting state and local government teams to own the RAISE tool has improved their understanding of adaptive program management and led to positive changes.

We describe key lessons learned and observations from implementing the tool and how challenges have been addressed.

### Make Context-Relevant Adaptations to the Implementation Process to Maximize Benefit

According to a review of 91 organizational capacity assessment tools,^21^ the tools that have been found to be most effective are those that have been adapted or custom-designed for a particular organization and its context and needs. The authors of another article that evaluated the methodologies of organizational capacity assessment tools argued “that reliable and replicable tools that allow longitudinal assessment of capacity enable the most rigorous assessment of organizational development,” yet the authors also noted the important balance that must be struck to avoid creating a burden on the organization to conduct the assessment regularly.^25^ Krause et al. concluded that ideally, an organization would be able to develop more advanced self-assessment skills over time as a supplement to regular external assessments.^25^

Each state team made relevant adaptations to the implementation process based on their context to ensure they maximized the benefits from the assessment. Examples of these adaptations include the following.

#### Selection of Local Government Representatives to be Part of the Assessment

The maturity level of each state health system differs, with different agencies playing roles of varying prominence. However, all states ensure that the assessment team includes representatives that can enable a 360-degree view of the issues from the local and state government perspectives. Several states reviewed the makeup of their RAISE teams following the initial assessment to enable them to respond more accurately to specific indicators and plan effective interventions to close identified gaps. For instance, states that did not have the adolescent desk officer and finance officer present could not objectively answer the indicators related to adolescent health and funding indicators and, as such, made provisions for these staff members to join the team for subsequent assessment rounds.

#### Adjustment in Duration of the Assessment

Initially, the assessment took 3 days on average, with some of the states conducting an orientation workshop on the tool on day 1, actual assessment on day 2, and joint development of the remediation plan on day 3. Subsequent assessments have shortened in length, and the assessment can now be completed in 1–2 days.

#### Changes in Implementation Medium

Because the RAISE tool was introduced at the beginning of the COVID-19 pandemic, states used virtual approaches, primarily relying on Zoom, for the initial orientation and assessment and to disseminate the findings and action plan. However, most states have subsequently held in-person assessments, adhering to COVID-19 prevention measures and protocols. Implementing the RAISE assessment virtually requires more days to complete than if the assessment is conducted in person. This is attributed to participants' lower ability to concentrate for long periods of time on Zoom and challenges with Internet connectivity. Senior government officials were unable to participate in the early assessments due to competing COVID-19 demands, but the RAISE assessment team ensured they received feedback on the assessment outcomes during relevant management meetings.

#### Use of Different Platforms to Disseminate Results

Each state uses different dissemination platforms, including technical working group meetings, contraceptive technology updates where emerging issues about FP programming are discussed, and program management team meetings, to communicate and provide feedback on the results and action plans to the larger FP/RH team.

### Account for Dips in Scores After Initial Improvement

It is important to highlight the reduction occasionally seen in the results where states scored lower in subsequent rounds compared to a previous assessment. This is attributed mostly to data-related indicators where states were measured based on reporting rates (such as timeliness and completeness) for the quarter in review. For instance, states that fell short of optimal reporting rates or that didn't release funds for program implementation during the quarter being assessed scored lower compared to previous quarters when the states had excellent reporting rates and released more funds. This finding aligns with the use of organizational capacity assessment tools that have been implemented in complex government systems, given regular staff turnover and changes in positions. As a result, it has been argued that organizational capacity assessment tools should not be used for evaluative purposes but instead to facilitate a process of individual reflection and development of collective meaning that leads to group decision-making and action planning.^22^ The Table includes illustrative action plans to address these kinds of dips in the scores.

**TABLE:** Sample RAISE Action Plan

Major Gaps Identified	Planned Activities to Address Gaps	Responsible Persons
Inconsistent data at facility, local government, and state levels	Coaching of facility service providers and M&E officers on proper documentation and reporting Coaching of local government M&E officers on data validation and data quality	LGA HMIS officers State HMIS officer
Lack of funding for SBC interventions	Advocacy to key decision-makers to secure funding for SBC interventions Inclusion of key SBC interventions in state health plans	Director of public health State health educator
Weak community-to-facility referrals	Coaching of community and facility providers on strategies for achieving effective 2-way referrals Deployment of community social mobilizers to support client referrals and follow-up	LGA health educators Facility referral officer

Abbreviations: HMIS, health management information system; LGA, local government area; M&E, monitoring and evaluation; RAISE, Reflection and Action to Improve Self-reliance and Effectiveness; SBC, social and behavior change.

### System-Level Changes Take Considerable Time

Some indicators, such as policy adoption and domestication, lack of an FP budget line, institutionalization of proven interventions, and integrated commodity logistics systems for primary health care programs, that are measured by the RAISE tool are at a systems level and, as such, may take some time to change. These indicators often score low or fluctuate greatly depending on the time of the fiscal year and other national-level initiatives. As a result, the states have expressed a desire to change the frequency of the assessment from quarterly to semiannually. However, TCI Nigeria and the state governments have agreed to continue implementing the RAISE tool quarterly to ensure a comfort level with the tool and inform coaching needs for the first 2–3 years of engagement and then adjust to semiannually upon graduating from direct support from TCI.

### Build Capacity to Facilitate Scale-Up of RAISE

Over time, the TCI Nigeria team has built the capacity of state government teams to facilitate the use of the RAISE tool. Across all supported states, state government teams are trained facilitators of the RAISE assessment. Beyond the 13 states assessed in the study, in 2022, TCI expanded to an additional 8 new states (Osun, Kwara, Edo, Borno, Sokoto, Yobe, Akwa Ibom and Adamawa). These states started using the tool, hoping to learn how to improve their FP/RH programs through its utilization. All of these states have benefited from a baseline RAISE assessment and will be supported to continue the assessment quarterly with emphasis on the implementation of corrective actions developed to support improvements.

## CHALLENGES

Although the RAISE assessment contributed to improved responsiveness of governments to their FP/RH programs across focus states, there have been challenges with implementing and institutionalizing this RF mechanism and process. Challenges faced in introducing and implementing the RAISE tool are similar to the known obstacles in trying to promote a “test and learn” culture within well-established, hierarchical organizations, including acceptability, institutionalization, and scale-up.

### Ensuring Understanding and Buy-In of the Tool

In addition to the orientation conducted for key stakeholders at the state level, TCI Nigeria had to support the state FP coordinators to conduct one-on-one follow-up visits to specific influential stakeholders for better understanding, buy-in, and advocacy for the use of the tool.

### Accounting for Staff Attrition

Due to competing priorities and staff transfers, continuity of participating staff was often disrupted between assessment periods. As a result, new participants needed orientation before the assessment, which required additional planning and time. Inconsistency in scoring was often reported because of new participants conducting the assessment. For example, in Anambra state, the first assessment occurred after massive staff transfers that affected 80% of staff in the State Ministry of Health. These transfers resulted in significantly lower assessment scores than those in subsequent assessments after the new team members had become more familiar with the FP program and situation. In addition, a potential for bias exists with the scoring process, as with any other self-assessment tool. However, TCI tried to limit biases as much as possible by using a facilitated group assessment process and by triangulating the RAISE results with other data sources as a means of verification.

### Identifying a Facilitator

Institutionalization was also difficult, especially in the beginning, as some of the states reported the challenge of identifying a government facilitator to lead the process. Most of the states identified the state FP coordinator as lead facilitator because of the program focus, whereas a few states selected other staff within the unit as facilitators based on personal traits that enabled them to facilitate a consensus-building process. TCI Nigeria continues to strengthen the capacity of selected government facilitators and other interested staff, which is gradually leading to institutionalization of the assessment tool. Going forward—given their role as government watchdogs—TCI will advocate for representatives of civil society organizations who are members of the FP advocacy core groups across supported states to also join the assessment teams for real-time verification of scores in domains related to political commitment and funding.

TCI Nigeria continues to strengthen the capacity of selected government facilitators and other interested staff, which is gradually leading to institutionalization of the assessment tool.

### Scheduling Time for Assessments

Time is a major challenge across most of the TCI-supported states in Nigeria. State government team members are involved in multiple activities and sometimes find it difficult to dedicate time to the quarterly assessment. This has led to shifts in planned dates for the assessment, with some of the states missing the quarterly schedule and implementing it 4 or 5 months after the previous assessment. TCI has worked with state governments to try to reduce the shifts as much as possible through the inclusion of the RAISE assessment in routine state work plans and by sending early communication and advance reminders about the assessment date. TCI is currently working with the state government teams to ensure inclusion of the assessments in their annual operational plans.

## CONCLUSION

The RAISE tool has proven to be a valuable RF tool, effectively contributing to the enhancement of Nigerian state governments' leadership and management of their FP/RH programs. The approach is appreciated by state governments that are currently considering modifications to serve other integrated primary health care programs, such as malaria and nutrition. TCI will continue to monitor the RAISE scores alongside HMIS data across supported states, including states that have recently graduated from direct TCI assistance. This will enable TCI to track institutionalization of the tool, as well as its effectiveness in ensuring sustained program maturity and government capacity to implement the high-impact FP and AYSRH interventions. Government program managers, technocrats, and implementing partners should adopt the tool not only for monitoring and strengthening capacity but also as an RF tool for enabling adaptive management of development programs.

## Supplementary Material

GHSP-D-22-00189-supplement1.pdf

GHSP-D-22-00189-supplement2.pdf

## Notes

**Peer Reviewed**

**First published online:** March 6, 2023.
