# Supplementary material for: Improving State Government's Responsiveness to Family Planning Interventions in Nigeria Using an Innovative Reflection and Action Tool
Source: Glob Health Sci Pract. 2023 Dec 18;11(Suppl 2):e2200189. doi: 10.9745/GHSP-D-22-00189 (PMC10727459; doi:10.9745/GHSP-D-22-00189)
Supplement: GHSP-D-22-00189-supplement1.pdf [file GHSP-D-22-00189-supplement1.pdf]

Supplement to: Ajijola L, Igharo V, Anieto N, Mwaikambo L. Improving state government's responsiveness to family planning interventions in Nigeria using an innovative reflection and action tool. *Glob Health Sci Pract*. 2023;11(Suppl 2):e2200189.
https://doi.org/10.9745/GHSP-D-22-00189

THE CHALLENGE INITIATIVE
NIGERIA

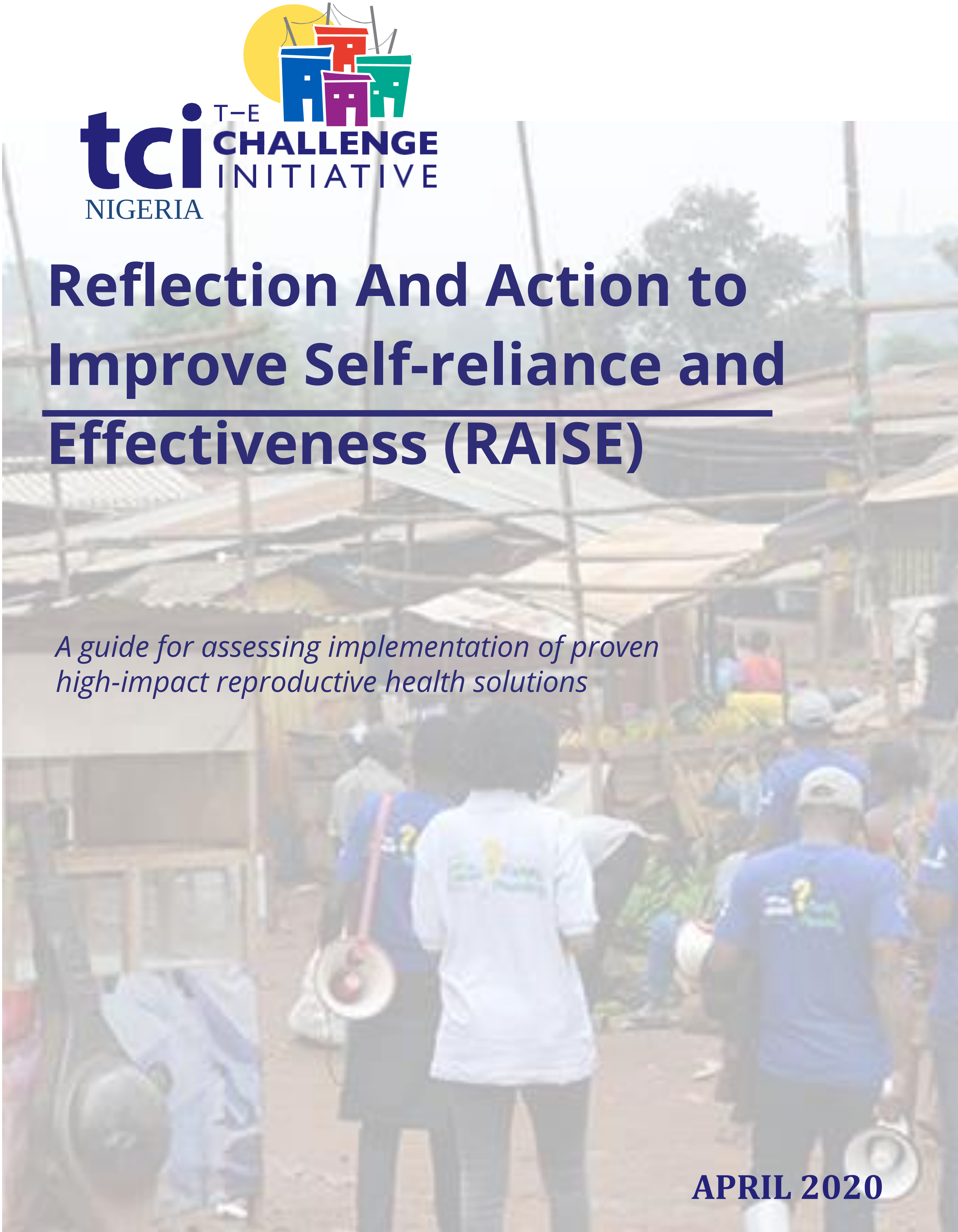

Reflection And Action to Improve Self-reliance and Effectiveness (RAISE)

A guide for assessing implementation of proven high-impact reproductive health solutions

APRIL 2020

TABLE OF CONTENTS

ACKNOWLEDGMENTS 1

ACRONYMS 2

BACKGROUND 3

RAISE Assessment Tool Overview 4

STATE GOVERNMENT RAISE ASSESSMENT PROCESS 6

Prospective Audience 6

Structure 6

Criteria 7

SCORING 8

Helpful Tips 9

WORKSHOP MATERIALS 11

RAISE Assessment Form 12

RAISE Assessment Consensus Form 27

State Government Action Plan Form 38

REFERENCES 40

// ACKNOWLEDGMENTS

This assessment tool was adapted from The Challenge Initiative (TCI) East Africa Hub. They would like to acknowledge and thank the following individuals for their valuable contributions to this document.

- Kojo Lokko, Executive Director TCI
- Paul Nychae, Regional Director TCI East Africa
- Allan Katamba Ssemakula, Country Lead TCI Uganda
- Peter Kagwe, Country Lead TCI Kenya
- Rose Mnzava, Country Lead TCI Tanzania
- Kathryn Walsh, Program Officer, TCI
- Kim Martin, Senior Program Officer, TCI
- Christina Cherel, Communications Associate, TCI
- Magdalene Mbondo, County Manager TCI Kenya
- Charles Mushi, M&E Manager TCI Tanzania
- Janet Adongo, Senior Technical Officer TCI Uganda
- Albert Bwire, AYRH Program Officer TCI Uganda
- Levis Onsase, County Manager TCI Kenya
- Assumpta Matekwa, County Manager TCI Kenya
- Kate Cho, Senior Program Officer, Advocacy & Accountability
- Health Department, Mukono Municipality, Uganda
- Health Department, Makindye Ssabagabo Municipality, Uganda
- Health Department, Buikwe District, Uganda
- Health Department, Kawempe Division, Kampala, Uganda
- Health Department, Nairobi County, Nairobi, Kenya
- Health Department, Kilifi County, Nairobi, Kenya
- Health Department, Migori County, Nairobi, Kenya

ACRONYMS

AOP	Annual Operational Plan
ACG	Advocacy Core Group
AYRH	Adolescent and Youth Reproductive Health
BMGF	Bill & Melinda Gates Foundation
CIP	Costed Implementation Plan
CLMS	Contraceptive Logistics Management Systems
CoP	Community of Practice
FP	Family Planning
HMIS	Health Management Information System
LARC	Long-acting Reversible Contraceptive
LG	Local government/s
LGA	Local Government Area
SBCC	Social and Behavior Change Communication
SDP	Service Delivery Point
SMoH	State Ministry of Health
SPHCDA	State Primary Health Care Development Agency
MDA	Ministries, Departments & Agencies
MSC	Most Significant Change
PIT	Project Implementation Team
RAISE	Reflection in Action to Improve Self Reliance and Effectiveness
TCI	The Challenge Initiative
TCI-U	TCI University
TWG	Technical Working Group

BACKGROUND

The Challenge Initiative (TCI) is a "business unusual" approach to financing, scaling up and sustaining reproductive health solutions among women 15-49 years of age in urban poor areas, with a recent additional emphasis on adolescents and youth, newlyweds and first-time parents 15-24 years of age.

TCI builds off evidence from the Bill & Melinda Gates Foundation's $150-million Urban Reproductive Health Initiative (URHI) by using URHI's proven solutions for implementation. It is a departure from the typical aid model because interested cities must bring their own resources and political will to the table to access funds from TCI.

TCI does not implement, but rather it works through regional "accelerator hubs" to provide ongoing coaching and technical guidance (Figure 1) to the cities and states as they implement proven interventions found on TCI University – an online platform for codifying, adapting, learning and sharing TCI's proven approaches.

TCI partners with four hubs – Jhpiego in East Africa, IntraHealth in Francophone West Africa, Johns Hopkins Center for Communication Programs (CCP) in Nigeria, and Population Services International (PSI) in India – to support local governments currently implementing. TCI Nigeria currently supports 78 local government areas (LGAs) across 10 states to implement family planning (FP) programs, with plans for expansion to additional LGAs and states in the coming years. It is expected that through technical assistance from TCI and with the state government taking ownership of implementation, family planning programs will be sustained in the country.

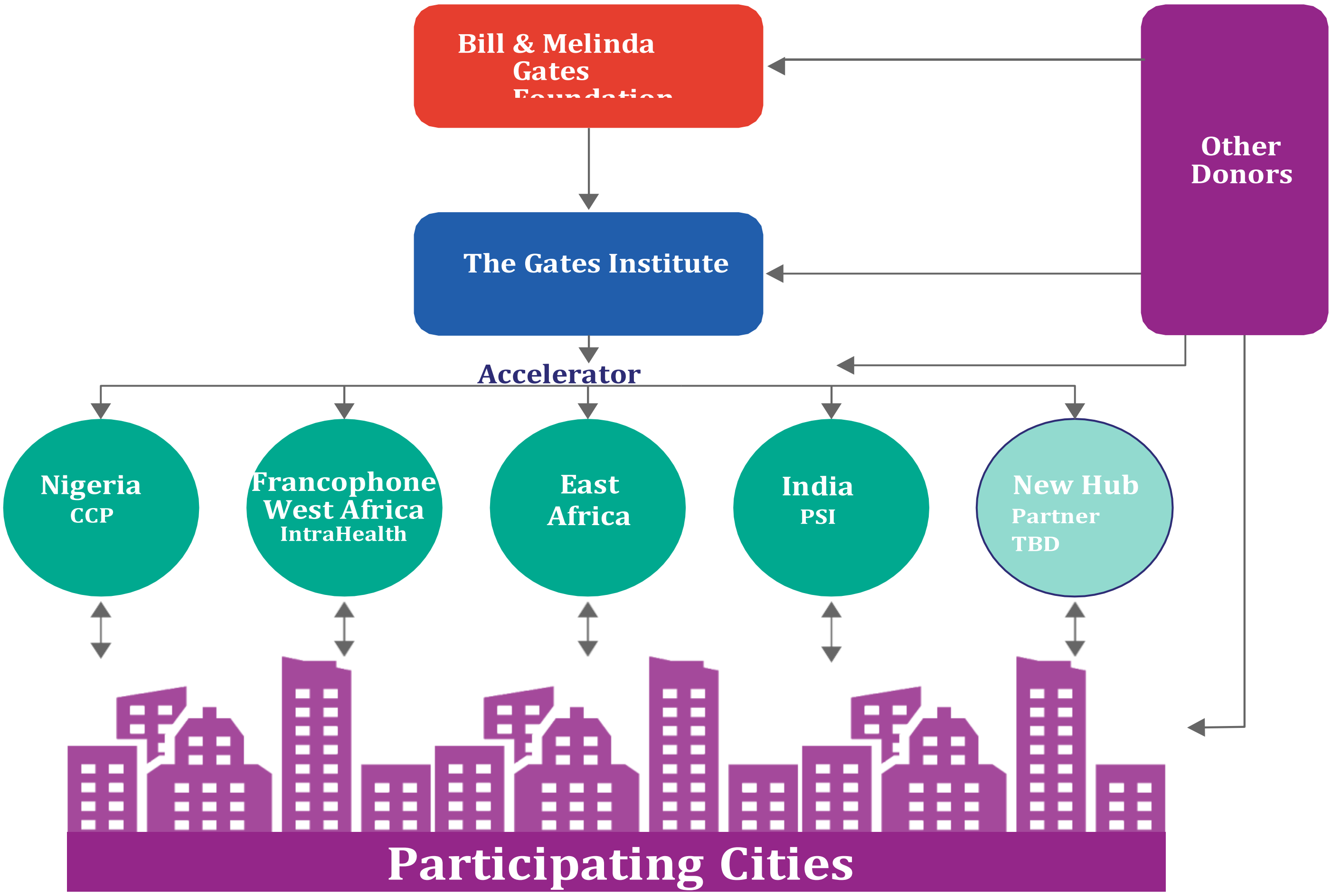

Figure 1: TCI's structure

TCI supports:

- State government buy-in and leadership and ownership of the program (including dedicated financial resources)
- Targeted technical assistance to the government to develop a program design for family planning with proven solutions, utilizing TCI-U
- Coaching of state government officials in program design and implementation for geographies to scale up tested family planning interventions

REFLECTION IN ACTION TO IMPROVE SELF RELIANCE AND EFFECTIVENESS ASSESSMENT TOOL OVERVIEW

This tool was adapted from TCI's East Africa hub to assess the quality and sustainability of FP high-impact interventions in each implementing city. Since TCI provides technical coaching to governments as they implement the high-impact approaches found on TCI University, the tool is meant to be used directly by government staff, in partnership with TCI.

Activities key to TCI's success in each city include measuring the intensity of implementation, implementing course corrective actions, maintaining quality during rapid scale-up, encouraging local ownership that leads to sustainability, and engaging stakeholders in decision-making in meaningful ways. The tool utilizes a standard set of indicators that consider all these elements and help governments reflect on their implementation progress in four main areas identified in TCI's Sustainable Scale-Up Pillars (Figure 2).

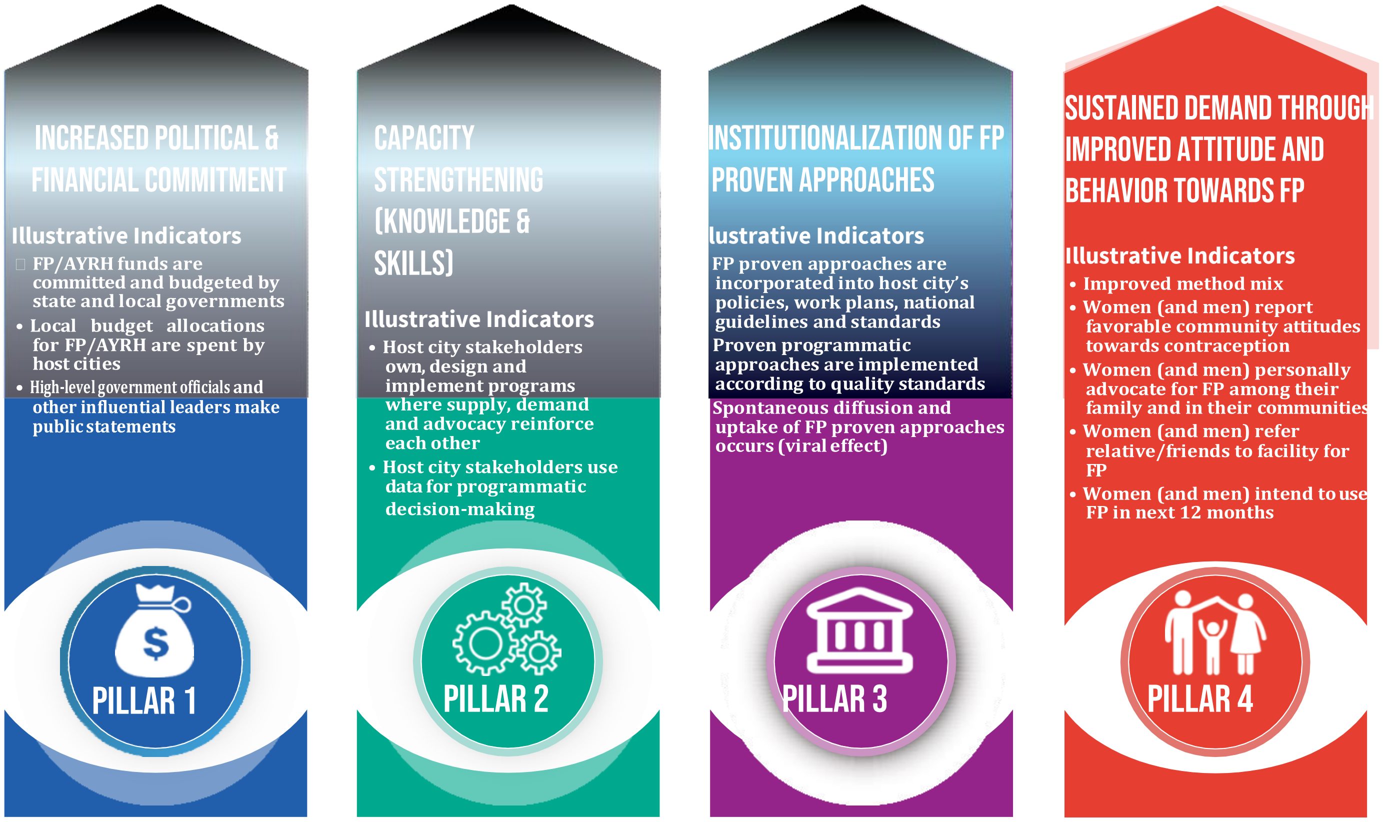

Figure 2: TCI's four sustainability pillars.

Through this reflective self-assessment tool, governments and their stakeholders:

1. Evaluate the quality of FP high impact interventions
2. Identify key areas in need of strengthening
3. Develop a concrete action plan for improvement
4. Monitor the progress of the action plan
5. Pinpoint program aspects that can serve as models for other states and government family planning programs

This tool promotes organizational learning, fosters an environment of best-practice sharing and enables state governments to own their improvement plans. By developing and implementing a systemic strategy, it will be easier for state governments to effectively assess and evaluate their family planning program interventions in a standardized manner and to recommend changes.

The tool is meant to be adaptable to other health areas outside of family planning and it is encouraged that governments (whether local, state or national) adapt the tool to their local needs. For instance, this version of the tool focuses on family planning, however, TCI plans to support government to adapt the tool to assess its adolescent and youth reproductive health (AYRH) interventions.

STATE GOVERNMENT RAISE ASSESSMENT PROCESS

PROSPECTIVE AUDIENCE

The tool was designed to be used by state and local governments that have (1) expressed interest in working with TCI, (2) recently started implementation of FP high-impact approaches, or (3) already implementing FP approaches for a period of time.

For governments new to TCI, the tool will help TCI determine the commitment of the government, the level of coaching and training needed, and relevant local partners. For governments already implementing FP approaches, it will help TCI gauge how the high-impact approaches have influenced the local government over time and the likelihood for sustainability when TCI's support (i.e., financial and coaching support) is eventually phased out. This will also help TCI to rank, recognize and reward governments, including eventually phasing-out or shifting its technical assistance from high-performing governments to those that may need more support.

This tool is intended to be used by program managers within the State Ministry of Health (SMoH)/ Department of Health and State Primary Health Care Development Agency (SPHCDA) that are responsible for implementing family planning programs. Ideally, the program managers would be part of a larger team working together with staff and leaders from all relevant departments in implementing states. At the very least, the following persons are recommended to participate in the assessment process:

- Available heads of relevant Ministries, Departments & Agencies (Honorable Commissioner for Health, Executive Secretary SPHCDA, Director of Public Health, Director of Community Health Services, Director of Pharmaceutical Services, Director of Budget, Director of Finance, Director of Planning, Research & Statistics etc.), Relevant Finance officer
- State and LGA RH/FP Supervisors
- State and LGA Monitoring & Evaluation Officers
- State & LGA Health Educators
- Adolescent Health Desk Officer
- Medical Officers of Health/ PHC Coordinators
- Representatives of ACG and other relevant TWGs (LMCU, RH TWG, SBCC TWG etc.)

STRUCTURE

The government self-assessment tool is meant to be used during an eight-hour workshop in one-day (9 am–5 pm). However, there is also pre-workshop preparation to make sure that the in-person meeting is productive and a post- workshop action plan with specified deliverables and timelines.

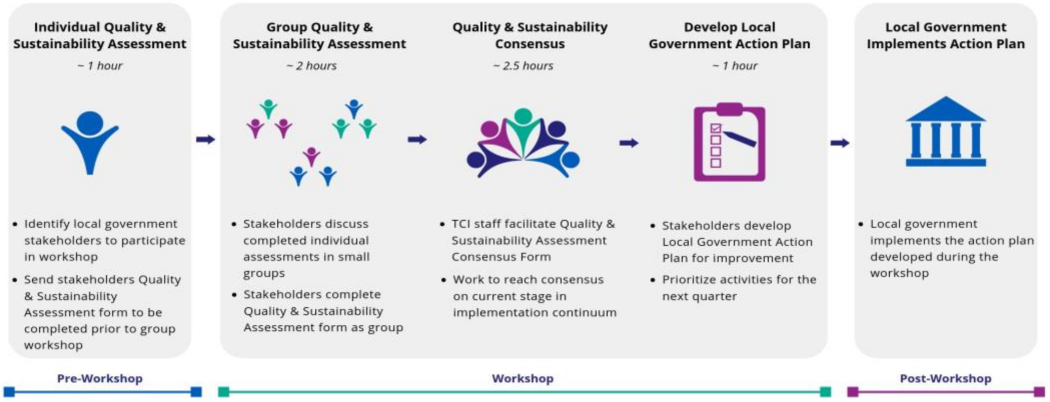

Figure 3: The process for conducting a TCI RAISE Assessment.

1. **Pre-workshop: Individual RAISE assessment tool review**
   In this step, the stakeholders in each state government needed to participate are identified and provided with the RAISE Assessment Form (page 12) to review and familiarize themselves with, prior to the group workshop. The main purpose of this individual exercise is to familiarize government staff with the tool, encourage self-reflection and ensure that attendees come prepared to the workshop.
2. **Workshop: Group RAISE consensus assessment**
   The one-day workshop (typically held quarterly) is structured into three main activities:
   - Small group work – Stakeholders first discuss their completed individual assessments in small groups and complete the same assessment form as a small group. This typically takes about two hours. TCI staff help guide the process of completing the RAISE Assessment Consensus Form (page 27).
   - Consensus – The small groups then reconvene for 2.5 hours to reach consensus on what stage they are in along the implementation continuum, noting relevant evidence.
   - Action planning – Stakeholders then develop a State Government Action Plan (page 38) for improvement over the last hour to prioritize activities for the next quarter
3. **Post-workshop: Implement Action Plan**
   After the workshop, the state government implement the action plan developed during the workshop.

Following the initial workshop, it is recommended that the state government organize quarterly workshops of the same format to evaluate progress, identify areas for improvements and develop a clear action plan for the next quarter. As states advance in implementation, these meetings may be held on a biannual or as-needed basis. At each subsequent meeting, TCI's role will lessen as government staff own the process and their assessment scores increase.

CRITERIA

This tool assesses capacity based on TCI's Sustainable Scale-Up Pillars (Figure 2) – focusing on the four pillars. Within each pillar, the tool utilizes the following criteria to assess local government capacity in family planning.

I. Increased Political and Financial Commitment

1 Leadership for FP & AYRH interventions
2. Policy and Guidelines for FP/AYRH program Implementation
3. Financial commitments
4. Financial spending
5. Financial management and documentation of funds

II. Capacity (Knowledge) Transfer of Family Planning and AYRH Skills

1. Improved Government Capacity for program implementation
2. Continuous quality improvement
3. Integration of Demand Generation and Service Delivery
4. Health Management Information Systems (HMIS) for FP/AYRH
5. Use of information for decision-making
6. Referral systems for FP/AYRH
7. Supportive supervision of interventions
8. Feedback and sharing of FP data and reports

III. Institutionalization of TCI's Proven Approaches at All Levels of the Health System

1. Access and utilization of TCI University
2. Coaching on FP/AYRH
3. Continuous adaptation of FP/AYRH high-impact approaches
4. Implementation of approaches according to quality standards
5. Diffusion of high-impact approaches
6. FP method mix
7. Integration of FP with other health services
8. Contraceptive procurement and logistics management
9. Public-private partnership
10. Community involvement in FP/AYRH

IV. Sustained Demand through Improved Attitudes and Behaviors towards FP & AYRH

1. Women (and men) report favorable community attitudes toward contraception
2. Women (and men) personally advocate for FP in their family and community
3. Women (and men) refer relatives/ friends to facility for FP/AYRH
4. Women (and men) intend to use FP in next 12 months
5. Proportion of modern method users shift toward LARC users from short-term methods users
6. Critical mass signifying demand for FP/AYRH
7. FP/AYRH outcomes sustained

SCORING

After each small group scores all the components under the four domains in the RAISE Assessment Form, they will compute the average score for each domain by adding the individual ratings and dividing by the number of components. For example, the first domain has seven components, so after rating each component from 1 to 4, add those ratings together and divide by 7.

At the end of the assessment, use the Level of Implementation computation form (Table 1) to get the final score. This is determined by weighting each domain and then computing an overall percentage. The criteria for each score are outlined in the tool itself (see page 12). It is important that supporting evidence is also provided with each score. This may include budgets, monitoring plans, meeting minutes and state government policy documents and reports. Where supporting evidence is not available, the state government entity should provide a justification for the score.

Table 1: Calculating the level of implementation progress

Capacity Domains	Average Score	Weight	Scoring
	Place your average score for each domain here.	*This signifies the importance, or "weight" assigned to each domain.*	*Multiply the average score by the weight*
**Domain 1**		30%	
**Domain 2**		20%	
**Domain 3**		25%	
**Domain 4**		25%	
		**TOTAL**	
		Average score *(total divided by 4)*	

The number in the purple box is the overall score and the state governments are ranked according the levels in Figure 4 below. Local governments that score 85-100% will be eligible for consideration to graduate since they have a high likelihood for long-term sustainability when TCI is eventually phased out. All state governments evaluated during the assessment period will be ranked based on their scores and the best performing will be rewarded in

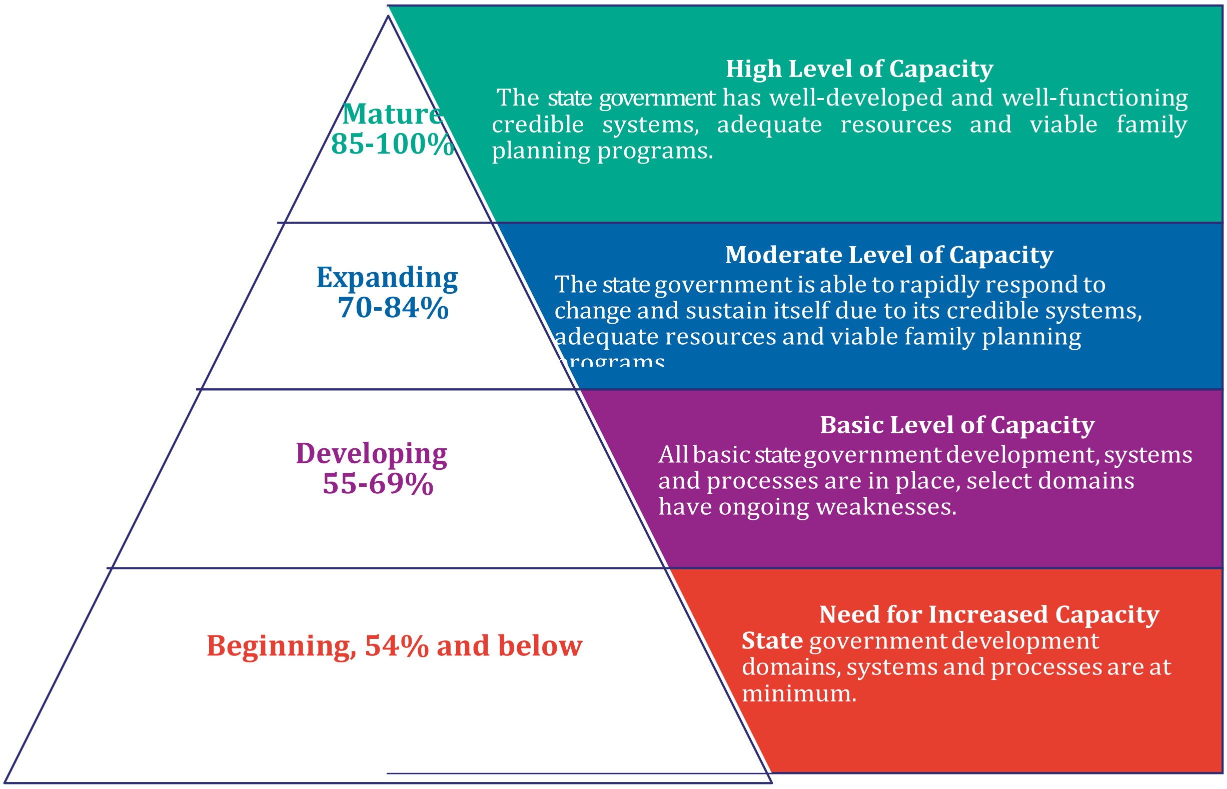

accordance with TCI's Recognition and Reward Strategy.

Figure 4: Four levels of performance are possible during the assessment.

HELPFUL TIPS

- State government leadership should be involved from the very beginning. Meet with necessary government officials early on to update them on the assessment tool's objective. Make sure they are well-aware of the process and give their concurrence to undertake the activity.
- TCI staff should facilitate the initial process. To ensure sustainability, TCI staff will progressively transfer facilitation skills to the state government so future assessments are conducted by local government staff with minimal TCI support.
- The tool is meant to be adaptable. If certain criteria are not applicable to a government or program, that remove or adapt it so that it makes sense.
- Agree with the relevant stakeholders on a list of participants, venue and date.
- Share an overview of the tool and the individual assessment form before the workshop with the proposed list of participants.
- Facilitators should ask open-ended, probing questions to encourage group discussion during the workshop, and take notes on participant responses. These notes are later used for action planning.
- The final scores are designed to set priorities for the actions and used to judge performance. The state government reviews or adjusts its performance and builds on the suggested actions to define next steps, responsibilities, time frame and possible technical assistance needs.

- TCI can plan its coaching based on which stage a government is in, identifying geographies that may need more intensive coaching and those that may need coaching to shift.
- Since this is work in progress, it is important to agree on a time for the next assessment workshop and to monitor the progress of action items and course corrections in the meantime.
- Be sure to tell participants that this assessment is not an end in itself, but, rather, one step in a significant change process, which requires all staff's commitment to implement the improvements in their day-to-day work.

WORKSHOP MATERIALS

1. RAISE Assessment Form (completed both individually prior to the workshop and as a small group during the workshop)
2. RAISE Assessment Consensus Form
3. State Government Action Plan Form

RAISE ASSESSMENT FORM

Individual Review

Each participant should be provided with the form in advance (either electronically or in hard copy) to familiarize themselves with the form and prepare answers to be deliberated within their small groups at the workshop. The duration for this exercise is about an hour.

Instructions:

- Please use the form to familiarize yourselves with the indicators measured to assess the state and local government's readiness to implement the family planning program
- Bring your individual assessment to the workshop for the next step of group work
- Ensure you document means of verification or evidence for scores given if applicable

Small group consensus exercise

The form will be completed during the meeting by small groups. The duration for this exercise is about two hours. For each capacity domain, group members should come to consensus on the level of development that best describes the state government, citing the evidence that all group members agree supports their decision. Record these in the far-right column of the table.

Instructions:

- Divide workshop participants into three groups per state government. Each small group should nominate one person to take notes on the RAISE Assessment Form as the other members discuss.
- Each group should identify the level of development for each capacity domain of their state government while providing examples from their experience, as evidence to support their assessment.
- Members of each group should discuss each capacity domain in turn, exploring any differences in their perceptions.
- Remember that everyone's viewpoint is equally valid because it represents that person's individual experience.
- All of the characteristics of a given development rating must be present to place the state government at that stage. If any single characteristic is absent, your local government fits an earlier stage.
- Circle the current level that best represents the status in your state government and indicate the evidence for that level.

Note: The RAISE Tool includes both family planning and AYRH programming together for each domain component. Users of the tool should assess and score the programs individually to ensure appropriate attention given that the state government's programming in one area may be stronger than the other.

RAISE ASSESSMENT FORM

Domain	Component	Criteria	Rating FP	Rating AY	Evidence	
**Domain 1: Political and Financial Commitment for FP/AYRH Intervention**						
**1.1**	**Leadership[1] for FP/AYRH interventions**<br><br>*Determine the capacity of the State's management to lead in the areas of FP/AYRH*	State has limited or no identified leadership, or committed members, for FP/AYRH issues.			**FP Evidence:**	**AY Evidence:**
		The State has an identified leadership (RH unit/AHD unit), or members, with a commitment to FP/AYRH issues.				
		The leadership, or committed members understand their roles and FP/AYRH issues.				
		The leadership, or committed members are providing strategic thinking and direction, planning, implementing and monitoring FP/AYRH interventions.				
**1.2**	**Policies and Guidelines for FP/AYRH program**<br>*Assess the ownership and design of FP/AYRH implementation strategies/ approaches*	State has no policy documents and guidelines (Costed Implementation Plan, Annual Operational Plan, FP/AYRH workplan) for implementing FP/AYRH programs.			**FP Evidence:**	**AY Evidence:**
		State has policy documents and guidelines articulating clear FP/AYRH strategies and approaches that was developed with full participation of all stakeholders including private sector and community				
		Evidence based FP/AYRH approaches are integrated into these policy documents and guidelines and are being used to guide FP/AYRH program implementation				
		FP/AYRH plans are being implemented by government with evidence of review and tracking of progress quarterly or as required				

[1] Leadership includes Project Implementation Team (PIT), Technical Working Groups, etc.

Domain	Component	Criteria	Rating		Evidence	
			FP	AY		
1.3	**Advocacy for FP/AYRH**<br>*Assess the extent of advocacy and engagement of policy makers to advance the FP/AYRH needs of the population.*	High-level government officials and other influential leaders are passively, or not advocating for FP/AYRH in the community.			**FP Evidence:**	**AY Evidence:**
		High-level government officials and other influential leaders are actively advocating for FP/AYRH in the community				
		High-level government officials and other influential leaders are making public statements at forums, public events and on the media. FP/AYRH visibility is high.				
		FP/AYRH issues and priority needs of the community are included in the agenda of high-level state meetings. State has adopted and/ or supported policies (laws, regulations, budgets, etc.) that advance FP/AYRH needs of the community.				
1.4	**State financial commitments to FP/AYRH intervention**<br>*Determine if the State commits funds for FP/AYRH interventions and this commitment increases each following financial year.*	State does not have FP/AYRH budget line and inconsistently commits and budget funds for FP/ AYRH interventions every Financial year.			**FP Evidence:**	**AY Evidence:**
		State has a budget line and consistently commits and budgets funds for FP/ AYRH interventions every financial year.				
		State financial commitment for FP/ AYRH interventions consistently increases every financial year.				
		State has multiple sources of funding for FP interventions like FP budget line, SOML, BHCPF, LGA Derivation fund etc.				
1.5	**Financial spending of State on FP/AYRH interventions**<br>*Determine if State spends its budgeted allocations for FP/AYRH interventions, timely avails funds and tracks expenditure.*	State does not release its committed funds for planned FP/AYRH activities (for current or most recent FY) on schedule.			**FP Evidence:**	**AY Evidence:**
		State releases some of its own committed funds (less than 50% for planned FP/AYRH activities on schedule as planned.				
		State spends more than 50% of own budgeted allocation for planned FP/AYRH activities.				
		State releases and spend more than 70% of committed funds based on matching fund requirement for planned FP/AYRH activities				

Domain	Component	Criteria	Rating		Evidence	
			FP	AY		
**1.6**	**Financial documentation of FP/AYRH funds**<br>*Assess if record keeping is adequate and if financial files are audit ready*	State has limited or no written financial documentation procedures for budgeted FP/AYRH funds.			**FP Evidence:**	**AY Evidence:**
		State has financial documentation procedures and complete and appropriate financial documentation of health activities' funds (including FP/AYRH) are available.				
		State staff involved in implementation of FP/AYRH activities understand the financial documentation procedures and consistently adhere to them.				
		In addition, financial documentation files for health activities' funds (including FP/AYRH) are regularly updated, stored in a secure location and audited internally.				
**1.7**	**Financial management system of FP/AYRH funds**<br>*Assess if financial management system is accurate and reliable*	State has limited or no designate finance system and staff for managing health sector funds, including FP/AYRH.			**FP Evidence:**	**AY Evidence:**
		State has a designate finance system and staff for managing health sector funds, including FP/AYRH.				
		The finance system for managing FP/AYRH funds is consistently adhered to, known and understood by all relevant finance and implementing staff.				
		The State finance system managing FP/AYRH funds presents an accurate, complete picture of expenditures, revenue, and cash flow in relation to FP/AYRH program outputs and services.				
**Average score:** Total the ratings for all 7 components for FP and 7 components for AYRH and divide by 7 (FP) and 7 (AYRH)					**Domain Average score: (FP Average plus AY Average) divide by 2**	

Domain	Component	Criteria	Rating		Evidence	
			FP	AY		
**Domain 2: Capacity (Knowledge and Skills) Transfer**						
**2.1**	**Government Capacity for FP/AYRH programs**<br>*Assess government capacity to lead and drive program implementation in the areas of FP/AYRH*	State government team lack requisite capacity to lead and drive implementation of FP/AYRH programs.			**FP Evidence:**	**AY Evidence:**
		State government team has some capacity to lead and drive FP/AYRH programs but depends majorly on Implementing Partners for technical assistance				
		State government team has required capacity to lead and drive FP/AYRH programs with little technical assistance from Implementing Partners				
		State government team has full capacity to lead and drive FP/AYRH program implementation without support or TA from Implementing partners				
**2.2**	**Commitment to leading and coordinating change**<br>*Determine the extent to which the state is willing to create new institutional arrangements (new or innovative rules and ways of doing things) in order to effectively scale up proven FP/AYRH solutions*	State accepts accountability for its leadership roles and actions – as individuals and as part of a collective team			**FP Evidence:**	**AY Evidence:**
		State takes leadership in collaborative testing and implementation of innovative interventions or practices that will result in more equitable outcomes, more responsive institutions and more empowered citizens				
		State ensures that collaborative innovations, partnerships and initiatives have clear and measurable outcomes with the required capabilities and resources to demonstrate impact				
		State puts in place regular review, monitoring and evaluation processes – ensures				
**2.3**	**Facility-to-Facility Referral systems for FP/AYRH**<br>*Assess the state's ability to ensure provision of comprehensive FP/AYRH services to clients through effective facility-to-facility referral systems*	The state has limited or no community to facility and or inter-facility referral systems for FP and AYRH			**FP Evidence:**	**AY Evidence:**
		The state has functional FP/AYRH referral systems for both community to facility and inter-facility referrals				
		State staff and volunteers have been trained on how to make effective referrals and relevant tools are available in adequate quantities. Tools include age of client referred.				
		The state periodically evaluates/ verifies that services were received for all clients and specifically for youth ages 15-24. State collects any feedback from clients, including AYs. The approach is documented and updated and can be shared as a model/resource.				

Domain	Component	Criteria	Rating		Evidence
			FP	AY	
2.4a	**Supportive supervision (SSV) of FP interventions**<br>*Establish the effectiveness of the FP support supervision structure.*	There is limited or no supervisory plan and structure for FP implementation.			**FP Evidence:**
		A supervisory plan, structure and process exists for FP implementation that include regular (monthly or quarterly) supervisory visits for commodities/logistics, health promotion/community mobilization, and service delivery.			
		FP Supervisory tools (Supply/Demand/M&E) are available in adequate quantities and supervisors are trained on their use.			
		State conducts regular SSV to assess quality of facilities and recommendations from the SSV are used to improve FP services			
2.4b	**Adolescent & Youth Friendly Health Services**<br>*Determine if the state regularly assesses the quality of AYRH services provided at the facility-level*	State does not have an adolescent & youth friendly health services (AYFHS) checklist to monitor the quality of services provided at health facilities			**AY Evidence:**
		State has a youth-friendly services checklist based on national or global AYFHS standards. The checklist includes AYRH Whole Site Orientation			
		State uses the youth-friendly services checklist (within its QI/QA system) to assess health facilities on a pre-determined basis (quarterly, bi-annually, annually) and has determined which facilities can be considered AYFHS.			
		State uses the AYFHS checklist (within its QI/QA system) to prioritize quality improvement interventions in low-performing facilities and the sharing of knowledge/best practices from high-performing facilities.			
2.5	**Access and utilization of TCI University**<br>*Determine the level of access and utilization of TCI University by state staff*	Less than 50% of the total expected state staff are registered in TCI-U and have completed less than five online courses on TCI-U FP/AYRH proven approaches and received a certificate			**FP Evidence:** **AY Evidence:**
		Less than 50% of the total expected state staff are registered in TCI-U and have completed five or more online courses on TCI-U FP/AYRH proven approaches and received a certificate			
		More than 50% of the total expected state staff are registered in TCI-U and have completed five or more online courses on TCI-U FP/AYRH proven approaches and received a certificate			
		More than 50% of the total expected state staff are registered in TCI-U and have completed five or more online courses on TCI-U FP/AYRH proven approaches and received a certificate. Staff submit at least 3 posts per month on TCI-U COP			

Domain	Component	Criteria	Rating		Evidence	
			FP	AY		
**2.6a**	**Health Management Information Systems (HMIS) for FP/AYRH**<br><br>***Completeness**: In the last 12 months, what was the **highest** reporting rate? Specify which month it occurred.*	Less than 50% of facilities reported data on FP/AYRH			**FP Evidence:**	**AY Evidence:**
		50-70% of facilities reported data on FP/AYRH				
		70-90% of facilities reported data on FP/AYRH				
		More than 90% of facilities reported data on FP/AYRH				
**2.6b**	**Health Management Information Systems (HMIS) for FP/AYRH**<br><br>***Completeness**: In the last 12 months, what was the **lowest** reporting rate? Specify which month it occurred.*	Less than 50% of facilities reported data on FP/AYRH			**FP Evidence:**	**AY Evidence:**
		50-70% of facilities reported data on FP/AYRH				
		70-90% of facilities reported data on FP/AYRH				
		More than 90% of facilities reported data on FP/AYRH				
**2.6c**	**Health Management Information Systems (HMIS) for FP/AYRH**<br><br>***Timeliness**: What was the reporting rate in the reporting month prior to this assessment?*	Less than 50% of facilities reported data on FP/AYRH			**FP Evidence:**	**AY Evidence:**
		50-70% of facilities reported data on FP/AYRH				
		70-90% of facilities reported data on FP/AYRH				
		More than 90% of facilities reported data on FP/AYRH				
**2.6d**	**Health Management Information Systems (HMIS) for FP/AYRH**<br><br>***Accuracy**: Did HMIS Data Quality Audits (DQAs) take place as scheduled?*	There were no FP/AYRH DQA efforts			**FP Evidence:**	**AY Evidence:**
		FP/AYRH DQA efforts were ad hoc				
		FP/AYRH DQAs were regularly scheduled but not always conducted				
		FP/AYRH DQAs were regularly scheduled and conducted				
**2.7a**	**Conduct of FP/AYRH data review meetings**	No meetings were held to review FP/AYRH data and program progress			**FP Evidence:**	**AY Evidence:**

Domain	Component	Criteria	Rating FP	Rating AY	Evidence	
	Assess the existence of mechanisms for evidence-based problem/ opportunities identification and decision making (e.g., performance review meetings) in the state	Meetings to review FP/AYRH data and program progress were held on an ad hoc basis				
		Meetings to review FP/AYRH data and program progress were regularly scheduled but not always held				
		Meetings to review FP/AYRH data and program progress were regularly scheduled and held				
**2.7b**	**Representation in FP/AYRH data review meetings** *In the performance review meetings conducted in the state, was there a broad representation of people who produce and utilize data, including key decision makers?*	No meetings were held to review FP/AYRH data and program progress			**FP Evidence:**	**AY Evidence:**
		In meetings held, participants were mainly data producers and limited representation of data users				
		In meetings held, there was good representation of participants from both data producers and data users, but not key decision makers				
		In meetings held, there was good representation of participants from both data producers and data users, including key decision makers				
**2.7c**	**Quality of FP/AYRH data review meetings** *In the performance review meetings conducted in the state, were programmatic implications adequately discussed?*	No meetings were held to review FP/AYRH data and program progress			**FP Evidence:**	**AY Evidence:**
		In meetings held, programmatic implications were not discussed				
		In meetings held, programmatic implications were discussed, but not adequately				
		In meetings held, programmatic implications were adequately discussed				
**2.8**	**Coaching on FP/AYRH**	The state has inadequate, or no trained coaches with appropriate technical expertise to conduct coaching on FP/AYRH			**FP Evidence:**	**AY Evidence:**

Domain	Component	Criteria	Rating		Evidence	
			FP	AY		
	Assess the relevance and effectiveness of coaching conducted by the state	The state has adequate number of trained coaches with appropriate technical expertise and skills who conduct coaching and mentoring of staff and volunteers on FP/AYRH based on work plan (for planned coaching), or coaching request from staff (on-demand coaching). Guidelines and IEC materials are availed to coachees during coaching sessions.				
		Coachees' acquisition of new skills is reinforced after the coaching session has ended and areas of further support identified through action planning and setting of measures of progress.				
		Coaching activities are periodically evaluated for their relevance and effectiveness and curricular or tools are updated based on findings and identified additional learning needs.				
**2.9**	**Usage of data to inform decision-making**<br>*Assess the extent to which data is utilized in decision-making processes*	Important decisions on FP/AYRH were ***almost always*** made without first reviewing the relevant data			**FP Evidence:**	**AY Evidence:**
		Important decisions on FP/AYRH were ***usually*** made without first reviewing the relevant data				
		Important decisions on FP/AYRH were ***sometimes*** made without first reviewing the relevant data				
		Important decisions on FP/AYRH were ***rarely made*** without first reviewing the relevant data				
**Average score:** Total the ratings for all 14 components for FP and 14 components for AYRH and divide by 14 (FP) and 14 (AYRH)					**Domain Average score: (FP Average plus AY Average) divide by 2**	

Domain	Component	Criteria	Rating		Evidence	
			FP	AY		
**Domain 3: Institutionalization of TCI Proven Approaches at All Levels of the Health System**						
**3.1**	**Adaptation and adoption of FP/AYRH proven high impact approaches**<br>*Determine whether the state has adapted FP/AYRH proven high impact approaches*<br>AYRH HIAs are SP training on provider bias AYRH WSO, use of AYFHS checklist Inreaches/outreaches for AY, SBCC for AY	FP/AYRH proven high impact approaches (HIA) have not been incorporated into the state policies, or workplans, guidelines and standards.			**FP Evidence:**	**AY Evidence:**
		At least 1 to 2 FP/AYRH proven HIAs have been incorporated into the state policies, or workplans, guidelines and standards.				
		At least 3-5 FP/AYRH proven HIAs have been incorporated into the state policies, or workplans, guidelines and standards.				
		More than 5 FP/AYRH proven HIAs have been incorporated into the state policies, or workplans, guidelines and standards and reflect data-informed priorities in advocacy, supply & demand generation				
**3.2**	**Implementation of FP/AYRH proven high impact approaches according to quality standards**<br>*Determine if FP/AYRH proven high impact approaches are implemented according to quality standards and guidelines*	The state has no trained service providers available to implement high impact FP/AYRH approaches and interventions			**FP Evidence:**	**AY Evidence:**
		The state has trained service providers but not implementing high impact FP/AYRH approaches and interventions				
		FP/AYRH service providers, including volunteers, have been trained and oriented on the FP/AYRH guidelines and toolkits and are implementing the high impact interventions				
		A system exists to verify providers' compliance with the guidelines and to provide targeted on-site knowledge and skill updates as needed. Compliance monitoring reports are available.				
**3.3**	**Scale up and Diffusion of FP/AYRH proven high impact approaches**<br>*Determine existence diffusion and scale up of FP/AYRH proven high impact approaches*	FP/AYRH proven high impact approaches are not implemented by Non-TCI directly supported health facilities and communities			**FP Evidence:**	**AY Evidence:**
		FP/AYRH proven high impact approaches are implemented by health facilities and communities not directly supported by TCI-funded program				
		FP/AYRH proven high impact approaches are implemented by State implementing partners not directly supported by TCI-funded program				
		FP/AYRH proven high impact approaches are implemented by other neighboring states not directly supported by TCI-funded program.				

Domain	Component	Criteria	Rating		Evidence	
			FP	AY		
**3.4**	**Integration of FP/AYRH with other health services**<br><br>*Determine if service providers integrate FP/AYRH services with other health services*	No supported HFs provide FP/AYRH services integrated with at least one other health service (e.g. RI, HIV/ AIDS, etc.).			**FP Evidence:**	**AY Evidence:**
		Less than 50% supported HFs provide FP/AYRH services integrated with at least one other health service				
		50% or more supported health facilities provide FP/AYRH services integrated with at least one other health service				
		Providers at all service delivery points integrating FP/AYRH services have been trained to offer FP/AYRH services (information and counselling).				
**3.5**	**Contraceptive Logistics Management Systems (CLMS)**<br><br>*Determine that the state has effective contraceptive logistics management systems for FP/AYRH*	State has a basic or no system for ensuring commodity security including storage and distribution of contraceptives.			**FP Evidence:**	**AY Evidence:**
		State has an established CLMS that adequately plan for and forecasts current and future commodity needs. This system takes into consideration AY FP needs (e.g., condoms and EC).				
		Contraceptive supplies are stored in safe, secure places, protected from excessive heat, cold, and humidity. A functioning inventory system exists that records all incoming and outgoing stock. Staff have been trained to use the system.				
		Trained staff consistently use the supply system to forecast future requirements, ensure adequate stock with less than 10% of HFs providing FP/AYRH services reporting stock				
**3.6**	**Public-private partnership for FP/AYRH**<br><br>*Determine if the state has a mechanism for involving both the public and private sectors (Private Hospitals & PPMVs) in FP/AYRH program planning, implementation and monitoring*	Public-private sector meetings held irregularly or not at all.			**FP Evidence:**	**AY Evidence:**
		PPP sector meetings held regularly as scheduled, and all relevant private sector members are represented in these meetings.				
		During meetings, members share plans and review performance data, and use data to inform their decision-making.				
		All members feel ownership in taking action to advance FP/AYRH activities, including advocating for FP/AYRH in the community and there is coordination of efforts.				
**Average score:** Total the ratings for all 6 components for FP and 6 components for AYRH and divide by 6 (FP) and 6 (AYRH)					**FP Av + AY Av divide by 2**	

Domain	Component	Criteria	Rating FP	Rating AY	Evidence	
**Domain 4: Sustained Demand through Improved Attitudes and Behaviors towards FP**						
**4.1**	**Social Behavior Change Communication (SBCC) Management/Coordination** *Determine if the SBCC leadership has a vision, mission & ToR and coordinates partners and program implementation*	The state SBCC does not have vision, mission and terms of reference (ToR) to guide program implementation			**FP Evidence:**	
		The state SBCC has a vision and mission statement but no ToR to clarify roles and support functionality of the group				
		The state SBCC has a vision, mission and terms of reference (ToR) to guide committee activities but no strategy or leadership structure to coordinate partners and program implementation				
		A state SBCC with well-defined vision, mission, ToR, SBCC strategy with specific audience segments and approaches, and leadership structure to guide and coordinate program implementation.				
**4.2**	**State Policies and, or Structures Support Community engagement in FP/AYRH** *Determine the extent to which the state's policies and structures support community engagement in FP/AYRH programs*	The state does not have policies, or strategies and structures (including SBCC) to support community engagement in FP/AYRH programs			**FP Evidence:**	**AY Evidence:**
		The state has policies, or strategies and structures (including SBCC) for community engagement in FP/AYRH programs but not functional				
		The state has policies, or strategies and functional structures (including SBCC) focusing on community engagement in FP/AYRH programs				
		The State demonstrates willingness to revisit its policies, or strategies and structures to respond to community FP/AYRH needs and ideas. In addition, state provides funding for community engagement activities				
**4.3**	**FP method mix** *Determine that the state provides comprehensive FP method options to clients demonstrating method mix*	The state has limited contraceptive options for AYs and other women of reproductive age			**FP Evidence:**	**AY Evidence:**
		The state has less than 5 different modern contraceptive method options for AYs and other women of reproductive age.				
		The state has and offer different short term and long-term methods demonstrating FP method mix				
		A mechanism exists in the state to assess and ensure continuous FP method mix for women of reproductive age including AYs				

Domain	Component	Criteria	Rating FP	Rating AY	Evidence	
4.4	**Community-to-Facility Referral systems for FP/AYRH**<br><br>*Assess the state's ability to ensure provision of comprehensive FP/AYRH services to clients through effective community-to-facility referral systems*	The state has no documented community-to-facility[2] referral systems for FP/AYRH services available at all health service delivery points (health facilities and community)			**FP Evidence:**	**AY Evidence:**
		The state has documented community-to-facility referral systems for FP/AYRH services available at all health service delivery points (health facilities and community)				
		State Staff and volunteers (where applicable) have been trained on how to make effective referrals and relevant tools are available in adequate quantities. Tools include age of client referred.				
		The State periodically evaluates/ verifies that services were received for all clients and specifically for youth ages 15-24. LG collects any feedback from clients, including youth. The approach is documented and updated and can be shared as a model/resource.				
4.5	**Provider Behavior towards FP/AYRH**<br><br>*Determine if provider behavior supports improved access to comprehensive quality FP services to clients, including method mix*	There are no strategies in place to assess and address provider attitudes and bias towards FP/AYRH			**FP Evidence:**	**AY Evidence:**
		The state has some strategies in place to assess or address provider attitudes and bias towards FP/AYRH. Government conducts sessions on counselling youth on method choices but does not hold values clarification exercises for providers.				
		The state has trained FP/AYRH providers on Interpersonal Communication and Counseling. Governments conducts provider bias reduction sessions for AYRH that include values-clarification exercises for attitudinal change. However, government does not measure the effectiveness of these sessions.				
		The state has trained FP/AYRH providers on Interpersonal Communication and Counseling who are offering FP/AYRH services to clients. Government conducts provider bias reduction sessions for AYRH that include values-clarification exercises for attitudinal change. Government assesses results of these sessions.				

[2] Community-to-Facility referral system connects clients in the community to health facilities providing FP services not offered by the CHWs.

Domain	Component	Criteria	Rating FP	Rating AY	Evidence
**4.6**	**Community involvement and community-led FP/AYRH programs**<br>*Determine the extent to which the state's FP/AYRH programs reflect community needs and values, ensuring systematic demand generation*	The state provides limited or no opportunities for the community to participate in its FP/AYSRH activities.			**FP Evidence:**<br>**AY Evidence:**
		The state informs the community about its FP/AYSRH interventions.			
		The state involves community volunteers in FP/AYSRH program activities.			
		The state seeks broad community involvement and feedback in shaping FP/AYSRH program activities. FP/AYSRH leaders and organizations are engaged in designing and implementing FP/AYSRH programming.			
**4.7**	**Meaningful Involvement of young people in Adolescent Health Development**<br>*Determine if young people are meaningfully engaged in AYRH programming*	Government does not intentionally involve youth in decision-making processes.			**AY Evidence:**
		Government involves youth or youth-led organizations in some meetings. However, youth are not asked to contribute to the decision-making process and their feedback is not incorporated into policy/programs.			
		Government incorporates intentional youth engagement activities in annual workplan. Government involves youth or youth-led organizations in majority of regular government meetings and working groups. Youth voice their opinions and meaningfully contribute to program development.			
		Government has a youth engagement policy that requires youth participation in government meetings and program planning. Government tracks the number of meetings with youth participation and involves youth in RAISE tool assessments. Government partners with local youth organizations to identify and cultivate diverse group of youth leaders to be involved in AYRH program activities. There is a direct communication line between the young people, policy makers and program managers. Young people directly draft or are involved in drafting young people policy and program in the state.			

Domain	Component	Criteria	Rating		Evidence	
			FP	AY		
4.8	**Resource Mobilization and Utilization for Demand and SBCC Interventions**<br>*Determine if the state has funding for SBCC and demand interventions*	The state does not prioritize funding for demand and SBCC activities for FP/AYRH			**FP Evidence:**	**AY Evidence:**
		The state SBCC team has mapped sources of funding for demand and SBCC activities but lack skills to mobilize these resources for interventions				
		The state SBCC team has mapped sources of funding for demand and SBCC and equally have skills to mobilize resources for interventions but lack capacity for effective utilization				
		State has resource map, skilled in resource mobilization and has system for resource utilization on all SBCC interventions segmented as well for youth				
**Average score:** Total the ratings for all 7 components for FP and 7 components for AYRH and divide by 7 (FP) and 7 (AYRH)					**FP Av + AY Av divide by 2**	

Family Planning				
**Domain**	**Domain Average Score**	**Weighted score**	**Total Score**	**Grading**
Domain 1		30%		85-100% = Mature<br>70-84% = Expanding<br>55-69% = Developing<br><54% = Beginning
Domain 2		20%		
Domain 3		25%		
Domain 4		25%		
**Total**		**100%** / **4**		

AYRH				
**Domain**	**Domain Average Score**	**Weighted score**	**Total Score**	**Grading**
Domain 1		30%		85-100% = Platinum "Youth-Friendly City for AYRH" *<br>70-84% = Expanding "Youth-Friendly City for AYRH" *<br>55-69% = Developing city for AYRH<br><54% = Beginning city for AYRH
Domain 2		20%		
Domain 3		25%		
Domain 4		25%		
**Total Average**		**100%** / **4**		

RAISE ASSESSMENT CONSENSUS FORM

This form is completed in-person during the meeting. The duration for this exercise is 2.5 hours. For each capacity domain, the workshop members should come to consensus on the level of development that best describes the local government, citing the evidence that all workshop members agree supports their decision. Record these in the far-right column of the table

Instructions:

1. Place all workshop participants into one group representing their state government after they have completed their small group assessment.
2. The state government group should nominate one person to take notes on the Assessment Consensus Form as the members of each small group state the level of development, they chose for each capacity area, along with the evidence for that decision.
3. Use the central section of the form, under each group number, record the level of development that group selected.
4. In the larger white space beneath the group numbers and individual levels, summarize the evidence presented by all groups.
5. For each capacity domain, state government staff should come to consensus on the level of development that best describes their geography, citing one or two pieces of evidence that all members agree supports their decision. Record these in the far right column of the table.

Domain 1: Political and Financial Commitment for FP Intervention								
Component	Small Group Level Selected						State Consensus Level	
	FP			AY			FP	AY
	Group 1	Group 2	Group 3	Group 1	Group 2	Group 3		
1.1 Leadership for FP/AYRH Interventions								
	Small Group Evidence						Consensus Evidence	
Component	Small Group Level Selected						State Consensus Level	
	FP			AY			FP	AY
	Group 1	Group 2	Group 3	Group 1	Group 2	Group 3		
1.2 Policies and Guidelines for FP/AYRH programs								
	Small Group Evidence						Consensus Evidence	
Component	Small Group Level Selected						State Consensus Level	
	FP			AY			FP	AY
	Group 1	Group 2	Group 3	Group 1	Group 2	Group 3		
1.3 Advocacy for FP/AYSRH								
	Small Group Evidence						Consensus Evidence	

Component	Small Group Level Selected						State Consensus Level	
	FP			AY			FP	AY
	Group 1	Group 2	Group 3	Group 1	Group 2	Group 3		
1.4 State financial commitments to FP/AYRH interventions								
	Small Group Evidence						Consensus Evidence	
Component	Small Group Level Selected						State Consensus Level	
	FP						FP	
	Group 1	Group 2	Group 3	Group 1	Group 2	Group 3		
1.5 Financial spending of state on FP/AYRH interventions								
	Small Group Evidence						Consensus Evidence	
Component	Small Group Level Selected						State Consensus Level	
	FP			AY			FP	AY
	Group 1	Group 2	Group 3	Group 1	Group 2	Group 3		

**1.6 Financial documentation of FP/AYRH funds**	Small Group Evidence						Consensus Evidence	
Component	Small Group Level Selected						State Consensus Level	
	FP			AY			FP	AY
	Group 1	Group 2	Group 3	Group 1	Group 2	Group 3		
**1.7 Financial management system of FP/AYRH funds**								
	Small Group Evidence						Consensus Evidence	
**FP Average score:** (Total the State Consensus Levels for all 7 components for FP and divide by 7)		**AY Average score:** (Total the State Consensus Levels for all 7 components for AY and divide by 7)					**Domain 1 Average score:** (FP Average plus AY Average) divide by 2)	

Domain 2: Capacity (Knowledge and Skills) Transfer

Component	Small Group Level Selected						State Consensus Level	
	FP			AY			FP	AY
	Group 1	Group 2	Group 3	Group 1	Group 2	Group 3		
	Small Group Evidence						Consensus Evidence	

**2.1 Government Capacity for FP/AYRH interventions**								
Component	Small Group Level Selected						State Consensus Level	
	FP			AY			FP	AY
	Group 1	Group 2	Group 3	Group 1	Group 2	Group 3		
**2.2 Committed to leading and coordinating change**								
	Small Group Evidence						Consensus Evidence	
Component	Small Group Level Selected						State Consensus Level	
	FP			AY			FP	AY
	Group 1	Group 2	Group 3	Group 1	Group 2	Group 3		
**2.3 Facility to facility Referral Systems for FP/AYRH**								
	Small Group Evidence						Consensus Evidence	
Component	Small Group Level Selected						State Consensus Level	
	FP						FP	
	Group 1	Group 2	Group 3					

**2.4a Supportive Supervision for FP/AYRH Interventions**	Small Group Evidence						Consensus Evidence	
Component	Small Group Level Selected						State Consensus Level	
	AY						AY	
	Group 1	Group 2	Group 3					
**2.4b Adolescent and Youth Friendly Health Services**								
	Small Group Evidence						Consensus Evidence	
Component	Small Group Level Selected						State Consensus Level	
	FP			AY			FP	
	Group 1	Group 2	Group 3	Group 1	Group 2	Group 3		
**2.5 Access and utilization of TCI University**								
	Small Group Evidence						Consensus Evidence	
Component	Small Group Level Selected						State Consensus Level	

	FP			AY			FP	
	Group 1	Group 2	Group 3					
**2.6a Health Management Information Systems (HMIS) for FP/AYRH**<br>***Completeness: Highest reporting rate***								
	Small Group Evidence						Consensus Evidence	
**Component**	**Small Group Level Selected**						**State Consensus Level**	
	FP			AY			FP	AY
	Group 1	Group 2	Group 3	Group 1	Group 2	Group 3		
**2.6b Health Management Information Systems (HMIS) for FP/AYRH**<br>***Completeness: Lowest reporting rate***								
	Small Group Evidence						Consensus Evidence	
**Component**	**Small Group Level Selected**						**State Consensus Level**	
	FP			AY			FP	
	Group 1	Group 2	Group 3	Group 1	Group 2	Group 3		
**2.6c Health Management Information Systems (HMIS) for FP/AYRH**<br>***Timeliness***								
	Small Group Evidence						Consensus Evidence	

Component	Small Group Level Selected						State Consensus Level	
	FP			AY			FP	AY
	Group 1	Group 2	Group 3	Group 1	Group 2	Group 3		
**2.6d Health Management Information Systems (HMIS) for FP/AYRH**<br>***Accuracy***								
	Small Group Evidence						Consensus Evidence	

Component	Small Group Level Selected						State Consensus Level	
	FP			AY			FP	AY
	Group 1	Group 2	Group 3	Group 1	Group 2	Group 3		
**2.7a Conduct of FP/AYRH Data Review Meetings**								
	Small Group Evidence						Consensus Evidence	

Component	Small Group Level Selected						State Consensus Level	
	FP			AY			FP	AY
	Group 1	Group 2	Group 3	Group 1	Group 2	Group 3		
**2.7b Representation in FP/AYSRH Data Review Meetings**								
	Small Group Evidence						Consensus Evidence	

Component	Small Group Level Selected						State Consensus Level	
	FP			AY			FP	AY
	Group 1	Group 2	Group 3	Group 1	Group 2	Group 3		
2.7c Quality of FP/AYRH Data Review Meetings								
	Small Group Evidence						Consensus Evidence	

Component	Small Group Level Selected						State Consensus Level	
	FP			AY			FP	
	Group 1	Group 2	Group 3					
2.8 Coaching on FP/AYRH								
	Small Group Evidence						Consensus Evidence	

Component	Small Group Level Selected						State Consensus Level	
	FP			AY			FP	AY
	Group 1	Group 2	Group 3	Group 1	Group 2	Group 3		

2.9 Use of Data to inform Decision Making	Small Group Evidence		Consensus Evidence	

FP Average score: (Total the State Consensus Levels for all 14 components for FP and divide by 14)		AY Average score: (Total the State Consensus Levels for all 14 components for AY and divide by 14)		Domain 2 Average score: (FP Average plus AY Average) divide by 2)	

Domain 3: Institutionalization of TCI Proven Approaches at All Levels of the Health System

Component	Small Group Level Selected						State Consensus Level	
	FP			AY			FP	AY
	Group 1	Group 2	Group 3	Group 1	Group 2	Group 3		
3.1 Adaptation and adoption of FP/AYRH proven high impact approaches								
	Small Group Evidence						Consensus Evidence	

Component	Small Group Level Selected						State Consensus Level	
	FP			AY			FP	AY
	Group 1	Group 2	Group 3	Group 1	Group 2	Group 3		

**3.2 Implementation of FP/AYRH proven high impact approaches according to quality standards**								
	Small Group Evidence						Consensus Evidence	
Component	Small Group Level Selected						State Consensus Level	
	FP			AY			FP	AY
	Group 1	Group 2	Group 3	Group 1	Group 2	Group 3		
**3.3 Scale up and Diffusion of FP/AYRH proven high impact approaches**								
	Small Group Evidence						Consensus Evidence	
Component	Small Group Level Selected						State Consensus Level	
	FP			AY			FP	AY
	Group 1	Group 2	Group 3	Group 1	Group 2	Group 3		
**3.4 Integration of FP/AYRH with other health services**								
	Small Group Evidence						Consensus Evidence	
Component	Small Group Level Selected						State Consensus Level	
	FP			AY			FP	
	Group 1	Group 2	Group 3	Group 1	Group 2	Group 3		

3.5 Contraceptive procurement and logistics management	Small Group Evidence		Consensus Evidence	

Component	Small Group Level Selected						State Consensus Level	
	FP			AY			FP	AY
	Group 1	Group 2	Group 3					
3.6 Public-private partnership for FP/AYRH								
	Small Group Evidence						Consensus Evidence	

**FP Average score:** (Total the LG Consensus Levels for all 6 components for FP and divide by 6)		**AY Average score:** (Total the LG Consensus Levels for all 6 components for AY and divide by 6)		**Domain 3 Average score:** (FP Average plus AY Average) divide by 2)	

Domain 4: Sustained Impact through Improved Attitudes and Behaviors towards FP

Component	Small Group Level Selected			State Consensus Level
	FP			FP
	Group 1	Group 2	Group 3	
4.1 SBCC management and coordination				
	Small Group Evidence			Consensus Evidence

Component	Small Group Level Selected						State Consensus Level	
	FP			AY			FP	AY
	Group 1	Group 2	Group 3	Group 1	Group 2	Group 3		
4.2 State policies, and or structures support community engagement in FP/AYRH								
	Small Group Evidence						Consensus Evidence	

Component	Small Group Level Selected						State Consensus Level	
	FP			AY			FP	AY
	Group 1	Group 2	Group 3	Group 1	Group 2	Group 3		
4.3 FP method mix								
	Small Group Evidence						Consensus Evidence	

Component	Small Group Level Selected						State Consensus Level	
	FP			AY			FP	AY
	Group 1	Group 2	Group 3	Group 1	Group 2	Group 3		
4.4 Community to facility Referral Systems for FP/AYRH								
	Small Group Evidence						Consensus Evidence	

Component	Small Group Level Selected						State Consensus Level	

	FP			AY			FP	AY
	Group 1	Group 2	Group 3	Group 1	Group 2	Group 3		
**4.5 Provider Bias towards FP**								
	Small Group Evidence						Consensus Evidence	
Component	Small Group Level Selected						State Consensus Level	
	FP			AY			FP	AY
	Group 1	Group 2	Group 3	Group 1	Group 2	Group 3		
**4.6 Community Involvement in FP/AYRH**								
	Small Group Evidence						Consensus Evidence	
Component	Small Group Level Selected						State Consensus Level	
	AY						AY	
	Group 1	Group 2	Group 3					
**4.7 Meaningful Involvement of young people in Adolescent Health Development**								
	Small Group Evidence						Consensus Evidence	

Component	Small Group Level Selected						State Consensus Level	
	FP			AY			FP	AY
	Group 1	Group 2	Group 3	Group 1	Group 2	Group 3		
**4.8 Resource Mobilization and Utilization for Demand and SBCC Interventions**								
	Small Group Evidence						Consensus Evidence	
**FP Average score:** (Total the State Consensus Levels for all 7 components for FP and divide by 7)		**AY Average score:** (Total the State Consensus Levels for all 7 components for AY and divide by 7)					**Domain 4 Average score:** (FP Average plus AY Average) divide by 2)	

Family Planning and AYSRH				
**Domain**	**Domain Average Score**	**Weighted score**	**Total Average Weighted Score**	**Grading**
Domain 1		35%		85-100% = Mature<br>70-84% = Expanding<br>55-69% = Developing<br><54% = Beginning
Domain 2		25%		
Domain 3		25%		
Domain 4		15%		
**Total Average**		**100% / 4**		

STATE GOVERNMENT ACTION PLAN FORM

Using this form, the group identifies the gaps identified during the assessment (preference given to components that received a score of 1 or 2).

Instructions:

1. Make as many copies of this form as are needed to encompass all the improvement objectives in the action plan.
2. State government members sit together in one group. Using the findings in the Assessment Consensus Form, they identify areas that need strengthening or further development.
3. The information for the first two columns should be copied from the State Assessment Consensus Form.
4. State government members should develop objectives that will lead to improvement of their performance, as well as relevant activities.
5. A responsible focal point person should be identified and assigned to each objective
6. All activities should have a timeline for implementation
7. If the state government requires support from partners, it should be clearly indicated in the form.
8. The state government focal person responsible for RAISE should ensure that the action plan developed is implemented before the next assessment

REMEDIATION PLAN																	
Sub-component	Consensus Current Level	Current Gaps	Improvement Activities	Support Needed from Partners	State Staff Responsible	Timeline											
						Quarter 1			Quarter 2			Quarter 3			Quarter 4		
						Jan	Feb	Mar	Apr	May	Jun	Jul	Aug	Sep	Oct	Nov	Dec

REFERENCES

Civil Society Fund. (2011). *Organizational Capacity Assessment Tool*. Kampala, Uganda: Civil Society Fund.

Management Sciences for Health. (2010). *Management and Organizational Sustainability Tool: A Guide for Users and Facilitators*, 3rd edition. . Cambridge, MA 02139 USA.: Management Sciences feor Health.

New Partners Initiative Technical Assistance (NuPITA) Project. (2009). *Organizational Capacity Assessment Tool: Facilitator's Copy,* For Organizations Funded by USAID. Boston, MA 02210-1211: John Snow, Inc.

New Partners Initiative Technical Assistance (NuPITA) Project. (2014). *Technical Capacity Assessment (TCA) for Family Planning: Participant's Copy*. Boston, MA 02210-1211: John Snow, Inc.

The Challenge Initiative (unpublished report). (2019). Section III: *Pathways to Scale and Sustainability*. Baltimore, USA: Gates Institute.
