# Supplementary material for: Improving State Government's Responsiveness to Family Planning Interventions in Nigeria Using an Innovative Reflection and Action Tool
Source: Glob Health Sci Pract. 2023 Dec 18;11(Suppl 2):e2200189. doi: 10.9745/GHSP-D-22-00189 (PMC10727459; doi:10.9745/GHSP-D-22-00189)
Supplement: GHSP-D-22-00189-supplement2.pdf [file GHSP-D-22-00189-supplement2.pdf]

Supplement to: Ajijola L, Igharo V, Anieto N, Mwaikambo L. Improving state government's responsiveness to family planning interventions in Nigeria using an innovative reflection and action tool. *Glob Health Sci Pract*. 2023;11(Suppl 2):e2200189. https://doi.org/10.9745/GHSP-D-22-00189

SUPPLEMENT 2. RAISE Assessment Scores for States in Nigeria

TABLE S1. RAISE Assessment Scores in Group 1 States in Nigeria That Started Engagement With TCI in 2018

	RAISE Assessment Scores, %				
**States**	**Round 1**	**Round 2**	**Round 3**	**Round 4**	**Round 5**
Ogun	79[a]	83[a]	87[b]	88[b]	96[b]
Delta	82[a]	78[a]	80[a]	82[a]	87[b]
Kano	81[a]	77[a]	89[b]	95[b]	95[b]
Bauchi	80[a]	78[a]	85[b]	85[b]	93[b]
Niger	88[b]	91[b]	91[b]	91[b]	91[b]

Abbreviations: RAISE, Reflection and Action to Improve Self-reliance and Effectiveness; TCI, The Challenge Initiative.
[a] Expanding.
[b] Mature.

TABLE S2. RAISE Assessment Scores in Group 2 States in Nigeria That Started Engagement With TCI in 2019

	RAISE Assessment Scores, %				
**States**	**Round 1**	**Round 2**	**Round 3**	**Round 4**	**Round 5**
Anambra	68[a]	87[c]	88[c]	87[c]	88[c]
Plateau	80[b]	90[c]	90[c]	87[c]	88[c]
Abia	64[a]	76[b]	80[b]	74[b]	87[c]
Rivers	78[b]	77[b]	82[b]	84[b]	87[c]
Taraba	76[b]	79[b]	83[b]	84[b]	83[b]

Abbreviations: RAISE, Reflection and Action to Improve Self-reliance and Effectiveness; TCI, The Challenge Initiative.
[a] Developing.
[b] Expanding.
[c] Mature.

TABLE S3. RAISE Assessment Scores in Group 3 States in Nigeria That Started Engagement With TCI in 2021

	RAISE Assessment Scores, %				
**States**	**Round 1**	**Round 2**	**Round 3**	**Round 4**	**Round 5**
Lagos	78[a]	81[a]	83[a]	81[a]	78[a]
Nasarawa	69[b]	65[b]	63[b]	77[a]	82[a]
Gombe	65[b]	67[b]	68[b]	69[b]	74[a]

Abbreviations: RAISE, Reflection and Action to Improve Self-reliance and Effectiveness; TCI, The Challenge Initiative.
[a] Expanding.
[b] Developing.
